# Dietary Sulfur Amino Acid Sensing and Metabolic Adaptation: From the GCN2–ATF4–FGF21 Axis to Transsulfuration and Redox Homeostasis

**DOI:** 10.3390/nu18203382

**Published:** 2026-10-15

**Authors:** Roxana Liana Lucaciu, Adriana Corina Hangan, Lucia Maria Procopciuc

**Affiliations:** 1 Department of Pharmaceutical Biochemistry and Clinical Laboratory, Faculty of Pharmacy, “Iuliu-Hațieganu” University of Medicine and Pharmacy, 400349 Cluj-Napoca, Romania; liana.lucaciu@umfcluj.ro; 2 Department of Inorganic Chemistry, Faculty of Pharmacy, “Iuliu-Hațieganu” University of Medicine and Pharmacy, 400012 Cluj-Napoca, Romania; 3 Department of Medical Biochemistry, “Iuliu-Hațieganu” University of Medicine and Pharmacy, 400349 Cluj-Napoca, Romania; lprocopciuc@umfcluj.ro

**Keywords:** sulfur amino acids, methionine, cysteine, sulfur amino acid restriction, GCN2–eIF2α–ATF4, FGF21, SAMTOR–mTORC1, redox homeostasis, metabolic adaptation, precision nutrition

## Abstract

Sulfur amino acids (SAAs), mainly methionine and cysteine, play an important role in coordinating nutrient sensing, one-carbon metabolism, redox regulation, and whole-body energy homeostasis. Dietary methionine restriction (MR) and sulfur amino acid restriction (SAAR) have gained increasing interest as nutritional interventions that produce pronounced metabolic benefits in preclinical models, including reductions in adiposity and hepatic lipid accumulation and improvements in insulin sensitivity and glucose regulation. Human intervention studies suggest biological activity and potential metabolic benefit, although the available clinical evidence remains limited and heterogeneous. These effects arise from the coordinated activation of multiple nutrient-responsive pathways. Reduced amino acid availability can stimulate the general control nonderepressible 2–eukaryotic translation initiation factor 2 alpha–activating transcription factor 4 signaling pathway (GCN2–eIF2α–ATF4) integrated stress response, whereas changes in methionine-derived S-adenosylmethionine modulate mechanistic target of rapamycin complex 1 (mTORC1) activity through S-adenosylmethionine sensor upstream of mTORC1 (SAMTOR)-dependent signaling. At the systemic level, hepatic fibroblast growth factor 21 contributes to the regulation of adipose tissue remodeling, energy expenditure, thermogenesis, and insulin sensitivity. At the same time, alterations in transsulfuration reshape cysteine utilization, glutathione metabolism, hydrogen sulfide signaling, and mitochondrial redox adaptation. Importantly, selective MR, broader SAAR, and total protein restriction should not be considered equivalent interventions, as their metabolic effects are influenced by cysteine availability and the degree of restriction. Nevertheless, the translation of these effects to humans remains incompletely established, and available intervention studies are limited in number, duration, and sample size. This review examines the biochemical and molecular mechanisms underlying SAA sensing and metabolic adaptation and evaluates their potential relevance to obesity, type 2 diabetes, metabolic syndrome, and metabolic dysfunction-associated steatotic liver disease, with particular emphasis on future applications in precision nutrition.

## 1. Introduction

Sulfur amino acids (SAAs), principally methionine and cysteine, occupy a distinctive position in intermediary metabolism because they function not only as substrates for protein synthesis but also as regulators of methyl-group transfer, redox homeostasis, nutrient sensing, and cellular adaptation. Methionine is an essential amino acid and the precursor of S-adenosylmethionine (SAM), the major cellular methyl donor, whereas cysteine contributes to the synthesis of glutathione (GSH), hydrogen sulfide (H_2_S), and other sulfur-containing metabolites. Through the methionine cycle and transsulfuration pathway, the availability of these amino acids therefore links dietary protein intake with epigenetic regulation, antioxidant defense, and metabolic signaling [1,2]. Methionine restriction (MR) is consequently increasingly viewed as a metabolic intervention rather than simply a reduction in the supply of an essential amino acid.

Preclinical studies, particularly in rodent models, have consistently shown that dietary MR or broader sulfur amino acid restriction (SAAR) can reduce adiposity, increase energy expenditure, improve insulin sensitivity and glucose homeostasis, and decrease hepatic lipid accumulation. Human intervention studies, although considerably fewer and generally of shorter duration, support the biological activity of SAA restriction but have demonstrated more modest and heterogeneous metabolic effects. These responses are often observed without proportional reductions in energy intake, indicating an active metabolic adaptation to sulfur amino acid scarcity [2,3]. Importantly, recent work has also emphasized that methionine and cysteine restriction are not biologically equivalent and that cysteine availability can independently modify lipid metabolism, redox balance, and the overall response to SAAR. Thus, the metabolic phenotype depends not only on methionine intake but also on the total availability and relative proportion of dietary SAA.

At the cellular level, reduced SAA availability engages interconnected nutrient-sensing pathways, including the integrated stress response and SAM-dependent mTORC1 signaling, which subsequently coordinate endocrine and metabolic adaptation [4,5,6].

At the systemic level, hepatic FGF21 contributes to changes in energy expenditure, adipose tissue remodeling, glucose regulation, and substrate utilization, although not all responses to SAA restriction are FGF21-dependent [7].

SAA availability also influences transsulfuration, glutathione metabolism, H_2_S signaling, and redox homeostasis, linking dietary restriction to both adaptive responses and, when excessive, the risk of functional deficiency [2]. Nutritionally controlled SAA restriction may activate adaptive redox responses and improve stress resistance, whereas excessive sulfur amino acid depletion can compromise essential thiol-dependent processes. The quantitative distinction between controlled and more severe restriction is discussed in Section 8.3.

The biological consequences of restriction are therefore strongly dose-dependent, raising the important concept of a metabolic window in which nutrient stress promotes adaptation without progressing to functional deficiency [2].

Although much of the mechanistic evidence derives from experimental models, human studies increasingly support the biological relevance of SAAR. Controlled interventions have shown that restriction of methionine and cysteine can modify circulating FGF21 and sulfur metabolites, while an eight-week randomized trial in adults with overweight or obesity demonstrated greater weight loss, reduced leptin, and increased ketone bodies with SAAR compared with a higher-SAA diet [8,9]. More broadly, controlled protein restriction in healthy lean men has recently been shown to increase circulating FGF21 and the energy requirement necessary to maintain body weight, providing further evidence that amino acid availability influences human energy metabolism [10].

Despite these advances, the literature remains fragmented across nutrient sensing, methionine metabolism, endocrine signaling, transsulfuration, and redox biology. Moreover, MR, SAAR, and total protein restriction are frequently discussed together despite important mechanistic and nutritional differences. The aim of this review is therefore to integrate current evidence linking dietary sulfur amino acid availability with the GCN2–eIF2α–ATF4–FGF21 axis, SAMTOR–mTORC1 signaling, transsulfuration, redox homeostasis, and systemic metabolic adaptation, while critically evaluating their implications for obesity, insulin resistance, type 2 diabetes mellitus (T2DM), metabolic syndrome, and metabolic dysfunction-associated steatotic liver disease (MASLD) and their potential translation into precision nutrition.

## 2. Methodology

This article was designed as a narrative review focused on the biochemical and molecular mechanisms underlying sulfur amino acid sensing and metabolic adaptation. Relevant literature was identified through searches of PubMed/MEDLINE and Scopus, with the final search performed in July 2026. Searches used combinations of terms including methionine restriction, sulfur amino acid restriction, cysteine, GCN2, ATF4, FGF21, SAMTOR, mTORC1, transsulfuration, glutathione, hydrogen sulfide, redox homeostasis, obesity, insulin resistance, type 2 diabetes, and metabolic dysfunction-associated steatotic liver disease. Representative search combinations included (“methionine restriction” OR “sulfur amino acid restriction” OR “cysteine restriction”) AND (“GCN2” OR “ATF4” OR “FGF21” OR “SAMTOR” OR “mTORC1”), as well as (“methionine restriction” OR “sulfur amino acid restriction”) AND (“obesity” OR “insulin resistance” OR “type 2 diabetes” OR “metabolic syndrome” OR “metabolic dysfunction-associated steatotic liver disease”).

Priority was given to original experimental studies, human intervention trials, and recent reviews published predominantly between 2015 and 2026, while older seminal studies were included when necessary to describe established biochemical pathways. English-language publications were considered. Studies were included when they were relevant to sulfur amino acid metabolism, nutrient-sensing mechanisms, redox regulation, or metabolic outcomes. Publications with limited relevance to the scope of the review, conference abstracts, editorials, commentaries, and duplicate publications were excluded.

Titles and abstracts were initially screened for relevance, followed by full-text evaluation of potentially relevant articles. Reference lists of selected original studies and recent reviews were also examined to identify additional pertinent publications. Studies were selected according to their relevance to sulfur amino acid metabolism, nutrient-sensing mechanisms, redox regulation, and metabolic outcomes.

The evidence was synthesized qualitatively, with particular emphasis on mechanistic integration, translational relevance, and differences between methionine restriction, total sulfur amino acid restriction, and broader protein restriction. Because this was a narrative rather than a systematic review, no formal systematic risk-of-bias assessment or quantitative evidence synthesis was performed.

## 3. Sulfur Amino Acid Metabolism: Biochemical Foundations

### 3.1. Dietary Sources and Physiological Roles of Methionine and Cysteine

Methionine and cysteine are the principal sulfur-containing amino acids relevant to human nutrition. Methionine is nutritionally indispensable, whereas cysteine is considered conditionally indispensable because it can be synthesized from methionine through transsulfuration. Dietary cysteine therefore exerts a methionine-sparing effect, reducing the amount of methionine that must be diverted toward endogenous cysteine synthesis [11,12].

Sulfur amino acid content varies according to dietary protein source, with many plant proteins providing lower total SAA exposure than animal-derived proteins, a feature that has been exploited in human SAAR interventions [8,12].

Beyond protein synthesis, methionine provides SAM for methylation reactions, whereas cysteine contributes to glutathione, H_2_S, and redox-sensitive signaling. These functions link dietary SAA availability to one-carbon metabolism, antioxidant defense, and metabolic regulation [12,13]. For clarity, the terminology used throughout this review is defined as follows. Selective methionine restriction (MR) refers to reduction in dietary methionine while cysteine intake is maintained; cysteine restriction refers to selective reduction in dietary cysteine/cystine without intentional methionine restriction; total sulfur amino acid restriction (SAAR) refers to simultaneous reduction in methionine and cysteine availability; and total protein restriction refers to reduction of overall dietary protein intake, thereby decreasing the availability of multiple amino acids rather than selectively targeting sulfur amino acids. These distinctions are particularly important because many experimental diets described as methionine-restricted contain little or no cysteine and may therefore function physiologically as total SAAR rather than as selective MR [11,12,13].

Thus, the response to SAA restriction depends on both methionine and cysteine availability, providing the biochemical basis for the nutrient-sensing mechanisms discussed below.

### 3.2. Methionine Cycle, SAM/SAH Balance and Methylation

The methionine cycle occupies a central position at the interface between SAA metabolism, one-carbon metabolism, and cellular methylation. Following cellular uptake, methionine is activated through an ATP-dependent reaction catalyzed by methionine adenosyltransferase (MAT), generating SAM. This reaction converts methionine into a metabolically activated sulfonium compound with a highly transferable methyl group, making SAM the principal methyl donor for a broad range of cellular methylation reactions. Consequently, intracellular methionine availability can directly influence methyl-group transfer, while fluctuations in SAM concentration provide an important metabolic link between dietary amino acid supply and the regulation of gene expression, chromatin state, protein function, membrane composition, and intermediary metabolism. Recent work has further emphasized that SAM concentrations are tightly controlled through multiple homeostatic mechanisms, reflecting the necessity of maintaining methyl-donor availability within a relatively narrow physiological range [14,15].

In mammals, SAM synthesis is mediated by MAT enzymes encoded primarily by methionine adenosyltransferase 1A (*MAT1A*) and methionine adenosyltransferase 2A (*MAT2A*). MAT1A is predominantly expressed in the adult liver, where methionine turnover and transmethylation flux are particularly high, whereas *MAT2A* is more widely expressed in extrahepatic tissues and proliferating cells. These isoforms differ in kinetic and regulatory properties and therefore contribute to tissue-specific control of SAM synthesis. The liver plays a particularly important role in whole-body methionine metabolism because it coordinates dietary methionine utilization, transmethylation, remethylation, and transsulfuration. Altered MAT expression or activity can therefore modify intracellular SAM availability and influence downstream metabolic and epigenetic processes. Experimental evidence indicates that perturbations in *MAT1A/MAT2A* expression can affect DNA methylation, lipid metabolism, oxidative stress, and proliferative signaling, underscoring the broader biological importance of maintaining appropriate SAM synthesis [15,16].

Once synthesized, SAM donates its methyl group to numerous acceptor molecules through reactions catalyzed by SAM-dependent methyltransferases. These reactions include methylation of DNA, histones, RNA, proteins, phosphatidylethanolamine, and a variety of small molecules. DNA methyltransferases transfer methyl groups from SAM to cytosine residues, principally at CpG dinucleotides, while histone methyltransferases modify lysine or arginine residues within histone tails. In this way, nutrient-dependent changes in SAM availability can potentially influence epigenetic regulation and transcriptional activity. SAM-dependent methylation also extends beyond classical epigenetics: RNA methylation, including N6-methyladenosine (m6A), protein methylation, and phospholipid methylation provide additional mechanisms through which methionine availability can regulate cellular phenotype [15,17,18]. The growing recognition that SAM homeostasis affects multiple layers of information processing has therefore expanded the concept of methionine metabolism from a simple biochemical cycle to a broader metabolic regulatory system.

Every SAM-dependent transmethylation reaction produces S-adenosylhomocysteine (SAH), which is subsequently hydrolyzed by S-adenosylhomocysteine hydrolase to adenosine and homocysteine. Importantly, SAH is not merely an inert metabolic product. It is a potent product inhibitor of many SAM-dependent methyltransferases and therefore exerts a major regulatory influence on methylation reactions. For this reason, cellular methylation capacity depends not solely on the absolute amount of SAM but on the balance between SAM and SAH. The SAM/SAH ratio is frequently referred to as the methylation potential or methylation index, with higher ratios generally favoring methyl-transfer reactions and lower ratios indicating reduced methylation capacity [19,20].

The interpretation of the SAM/SAH ratio, however, requires some caution. Experimental evidence has shown that an apparently similar reduction in the SAM/SAH ratio can have different biological consequences depending on whether it results primarily from decreased SAM or from accumulation of SAH. Because SAH directly inhibits methyltransferases, increases in intracellular SAH may have particularly strong effects on methylation reactions. Earlier mechanistic studies demonstrated that reduced methylation capacity correlated more closely with SAH accumulation than with SAM depletion alone, highlighting SAH as an important determinant of cellular methylation status [21]. This concept remains relevant in contemporary studies in which elevated SAH and reduced SAM/SAH ratios are associated with impaired methylation potential in metabolic and cardiovascular contexts [20,22].

The SAM/SAH balance is closely connected to homocysteine metabolism. Hydrolysis of SAH is reversible and thermodynamically favors SAH formation unless its products, adenosine and homocysteine, are efficiently removed. Consequently, accumulation of homocysteine may shift the reaction toward SAH synthesis, thereby reducing the SAM/SAH ratio and suppressing methylation capacity. This establishes a mechanistic link between disturbances in homocysteine metabolism and altered epigenetic or metabolic regulation. Nutritional factors that influence one-carbon metabolism, particularly folate, vitamin B12, vitamin B6, choline, and betaine, can therefore indirectly modulate cellular methylation capacity by affecting methionine regeneration and homocysteine disposal [23,24].

The dependence of methylation reactions on methionine-derived SAM provides a direct biochemical mechanism through which nutrition can influence epigenetic regulation. DNA and histone methylation are dynamic processes that contribute to the regulation of chromatin accessibility and gene expression, and both require SAM as a methyl-group donor. Changes in methionine availability, one-carbon metabolism, or SAM homeostasis may therefore influence epigenetic patterns, although the relationship is not necessarily linear. Cellular systems actively buffer fluctuations in SAM, and different methyltransferases have distinct affinities for SAM and sensitivities to SAH inhibition. As a result, some methylation reactions may be more responsive than others to changes in dietary methionine or intracellular methyl-donor availability [14,17]. This biochemical selectivity may explain why nutritional interventions can induce locus-specific or pathway-specific epigenetic responses rather than uniform global methylation changes.

Beyond its canonical role as a methyl donor, intracellular SAM also participates in metabolic regulatory mechanisms that help coordinate methionine availability with cellular homeostasis [18].

Maintaining SAM homeostasis is therefore particularly important under conditions of SAAR. A reduction in dietary methionine can lower substrate availability for SAM synthesis, but cellular responses are shaped by compensatory mechanisms involving MAT activity, remethylation, reduced methyl-group consumption, and changes in downstream metabolic flux. This adaptive response may help preserve essential methylation reactions while simultaneously modifying anabolic and stress-responsive signaling. Recent analyses of SAM homeostasis emphasize that cells actively regulate SAM synthesis, utilization, compartmentalization, and degradation to avoid both deficiency and excessive accumulation [14]. Excessive or prolonged disruption of this balance, however, may compromise methylation reactions and alter metabolic, epigenetic, or redox homeostasis.

### 3.3. Homocysteine Partitioning Between Remethylation and Transsulfuration

Homocysteine is a central intermediate in SAA metabolism and can follow two principal metabolic routes: remethylation, which regenerates methionine, and transsulfuration, which irreversibly directs sulfur toward cysteine synthesis. The relative flux through these pathways depends on methionine availability, methylation demand, cofactor status, and intracellular SAM concentrations [25,26,27].

The major remethylation pathway is catalyzed by methionine synthase, which uses 5-methyltetrahydrofolate as the methyl donor and vitamin B12 as an essential cofactor. This reaction links homocysteine metabolism to the folate-dependent one-carbon cycle [23,26].

An alternative pathway, active mainly in the liver and kidney, is catalyzed by betaine-homocysteine methyltransferase (BHMT) and uses betaine derived from diet or choline metabolism as the methyl donor [26,28].

The transsulfuration pathway converts homocysteine to cysteine through two sequential reactions. Cystathionine β-synthase (CBS) condenses homocysteine with serine to form cystathionine, which is subsequently cleaved by cystathionine γ-lyase (CSE; encoded by *CTH*) to produce cysteine. These enzymes require pyridoxal-5′-phosphate (PLP) and therefore depend on adequate vitamin B6 availability [25,27]. Unlike remethylation, transsulfuration represents an irreversible loss of methionine-derived sulfur from the methionine cycle.

A major regulatory mechanism is provided by SAM. High intracellular SAM activates CBS and inhibits methylene tetrahydrofolate reductase (MTHFR), thereby favoring transsulfuration and cysteine production. Conversely, low SAM relieves MTHFR inhibition and reduces CBS activation, promoting remethylation and methionine conservation [25,27]. This reciprocal regulation allows SAA metabolism to adapt efficiently to changes in methionine availability.

These pathways are particularly relevant during SAAR. Reduced methionine availability favors methionine conservation through remethylation, whereas dietary cysteine can further spare methionine by decreasing the need for endogenous cysteine synthesis. Therefore, the metabolic effects of MR depend strongly on concomitant cysteine intake.

Remethylation and transsulfuration determine whether homocysteine-derived sulfur is recycled toward methionine and SAM production or redirected toward cysteine synthesis. Their coordinated regulation integrates methionine availability with one-carbon metabolism and provides the biochemical basis for downstream sulfur-dependent pathways.

### 3.4. Cysteine Partitioning into Glutathione and Hydrogen Sulfide Pathways

Cysteine occupies a central position in SAA metabolism because it can be incorporated into proteins or directed toward the synthesis of several sulfur-containing metabolites, including GSH, taurine, coenzyme A-related compounds, and H_2_S [2,29]. Among these metabolic fates, GSH synthesis and H_2_S production are particularly relevant to SAAR because both depend on cysteine availability and connect sulfur metabolism with cellular defense and signaling.

GSH is a tripeptide composed of glutamate, cysteine, and glycine and represents one of the major intracellular thiol-containing metabolites. Its synthesis occurs through two ATP-dependent reactions. First, glutamate and cysteine are condensed by glutamate-cysteine ligase (GCL) to form γ-glutamylcysteine, followed by addition of glycine through glutathione synthetase (GSS) [30]. Because intracellular cysteine availability can limit GSH synthesis, changes in cysteine supply directly influence the capacity to maintain intracellular glutathione pools.

Cellular cysteine is obtained through dietary uptake, reduction in extracellular cystine following transport through systems including system Xc^−^, or endogenous synthesis through transsulfuration [2,30]. These pathways provide metabolic flexibility when extracellular cysteine availability is reduced. During SAAR, preservation of cysteine becomes especially important because cysteine must be distributed among protein synthesis, glutathione production, and other sulfur-dependent pathways.

Through its cysteine thiol group, GSH participates in peroxide detoxification and maintenance of intracellular thiol homeostasis. Glutathione peroxidases use GSH as an electron donor to reduce hydrogen peroxide and lipid hydroperoxides, producing glutathione disulfide (GSSG), which can subsequently be reduced back to GSH using nicotin amid adenin dinucleotide phosphate (NADPH) [30,31].

Cysteine is also an important precursor of H_2_S, an endogenous signaling molecule generated primarily through CBS, CSE, and 3-mercaptopyruvate sulfur transferase (3-MST; encoded by *MPST*)-dependent pathways [29,32]. The relative contribution of these enzymes differs among tissues and metabolic conditions, thereby linking H_2_S production to both cysteine availability and transsulfuration activity.

H_2_S participates in a broader network of reactive sulfur species and influences multiple cellular processes. However, its physiological effects depend strongly on concentration and tissue context. In the setting of SAAR, changes in SAA availability may alter the partitioning of cysteine between GSH synthesis, H_2_S production, and other metabolic pathways [2,33]. Therefore, changes in total cysteine concentration alone do not necessarily reflect the extent of downstream sulfur signaling.

Cysteine functions as a critical metabolic branch point connecting SAA availability with antioxidant defense and sulfur-dependent signaling. Its utilization for GSH and H_2_S production provides the biochemical basis for the more complex redox, signaling, and mitochondrial adaptations induced by SAAR.

## 4. Molecular Mechanisms of Sulfur Amino Acid Sensing

### 4.1. GCN2–eIF2α–ATF4 Signaling and the Integrated Stress Response

GCN2–eIF2α–ATF4 is a major cellular system for sensing amino acid insufficiency and initiating an adaptive integrated stress response (ISR). During amino acid deprivation, accumulation of uncharged transfer RNA (tRNAs) and disturbances in translation activate general control nonderepressible 2, encoded by *EIF2AK4*. In mammalian cells, GCN2 activation is additionally facilitated by interactions with stalled ribosomes and the ribosomal P-stalk, indicating that this kinase integrates both amino acid availability and translational stress [34,35].

Activated GCN2 phosphorylates eukaryotic initiation factor 2α (eIF2α) at Ser51, reducing formation of the eukaryotic initiation factor 2α (eIF2)–GTP–initiator tRNA ternary complex and thereby attenuating global protein synthesis. This response decreases amino acid consumption and energetic demand during nutrient insufficiency. At the same time, eIF2α phosphorylation selectively promotes translation of specific stress-responsive transcripts, particularly ATF4, through regulatory upstream open reading frames within the *ATF4* mRNA [36,37].

ATF4 subsequently induces a broad adaptive transcriptional program involving amino acid transport, biosynthesis, autophagy, and stress resistance. Particularly relevant to SAA metabolism, ATF4 can increase expression of genes involved in cysteine acquisition and amino acid homeostasis, including solute carrier family 7 member 11 (*SLC7A11)*, thereby helping cells preserve intracellular cysteine availability under nutrient stress [4]. ATF4 also links cellular amino acid sensing to systemic metabolic adaptation by stimulating hepatic FGF21 expression during dietary amino acid or protein restriction [7,38].

However, the role of GCN2 in SAAR is more complex than in deprivation of some other essential amino acids. Leucine deprivation elicits a largely GCN2-dependent hepatic ISR, whereas SAA deprivation markedly increased *Atf4*, *Fgf21*, and amino acid transporter expression even in *Eif2ak4*-deficient mice [4]. These findings indicate that the hepatic response to methionine and cysteine deprivation can be maintained through GCN2-independent mechanisms.

Consistent with this concept, Wanders et al. showed that deletion of GCN2 did not abolish the characteristic metabolic responses to dietary methionine restriction, including increased energy expenditure, reduced adiposity, improved insulin sensitivity, and hepatic FGF21 induction [39]. In these animals, alternative signaling involving protein kinase RNA-like endoplasmic reticulum kinase (PERK) and nuclear factor erythroid 2-related factor 2 (NRF2) was activated, suggesting that SAA restriction can recruit additional stress-sensitive pathways that converge on eIF2α–ATF4 signaling.

Recent evidence further indicates that GCN2 can communicate with anabolic nutrient-sensing pathways beyond canonical eIF2α phosphorylation. The general control nonderepressible 2 (GCN2)–F-box only protein 22 (FBXO22) axis can promote mechanistic target of rapamycin (mTOR) ubiquitination during amino acid insufficiency, providing a direct molecular link between amino acid stress sensing and suppression of mTOR signaling [40]. This interaction complements the SAMTOR-dependent regulation of mTORC1 by methionine-derived SAM discussed in Section 3.2.

The GCN2–eIF2α–ATF4 pathway provides an important mechanism linking amino acid availability with translational control and metabolic adaptation. In the specific context of SAAR, however, GCN2 should not be considered an obligatory upstream regulator. Rather, ATF4 appears to function as a convergence point for multiple nutrient- and stress-sensitive pathways, enabling maintenance of the adaptive response even when canonical GCN2 signaling is absent.

### 4.2. Methionine and SAM Sensing Through the SAMTOR–mTORC1 Axis

mTORC1 is a central regulator of cellular growth and metabolism that integrates amino acid availability with growth-factor signaling, cellular energy status, and stress. Under nutrient-replete conditions, mTORC1 promotes protein, lipid, and nucleotide synthesis while suppressing autophagy. Methionine occupies a distinctive position within this regulatory network because its availability can be sensed indirectly through its metabolically activated derivative, SAM, thereby linking sulfur amino acid metabolism to anabolic signaling [5,41].

Amino acid-dependent regulation of mTORC1 occurs largely at the lysosomal surface. Ras-related GTPases (Rag GTPases) control recruitment of mTORC1 to lysosomes, where interaction with Ras homolog enriched in brain (Rheb) permits full kinase activation. Upstream, the GTPase-activating protein activity toward Rags 1 (GATOR1) complex, composed of DEP domain-containing protein 5 (DEPDC5), Nitrogen Permease Regulator 2 (NPRL2), and NPRL3, inhibits Rag-dependent mTORC1 activation during amino acid limitation, while KPTN–ITFG2–C12orf66–SZT2-containing regulator of mTORC1 (KICSTOR) anchors GATOR1 to the lysosomal membrane [41,42]. These components form a regulatory platform through which intracellular amino acid sensors modulate mTORC1 activity.

Methionine sensing is incorporated into this system through SAMTOR, a direct intracellular sensor of SAM. SAMTOR binds SAM and regulates mTORC1 according to methionine availability [5]. When methionine and intracellular SAM are limited, SAMTOR associates with the GATOR1–KICSTOR complex, enhancing inhibition of Rag-dependent mTORC1 signaling. Conversely, when SAM concentrations rise, SAM binding disrupts this inhibitory interaction and favors mTORC1 activation [5,6]. Thus, the sequence methionine → SAM → SAMTOR → GATOR1/KICSTOR → Rag GTPases → mTORC1 provides a direct biochemical mechanism connecting SAA availability with cellular anabolic activity.

Structural studies have strengthened this model. Disruption of SAM recognition alters mTORC1 regulation [6]. More recent cryo-electron microscopy of the KICSTOR–GATOR1–SAMTOR complex further clarified how SAMTOR is incorporated into the lysosomal nutrient-sensing machinery, supporting the concept that SAM availability determines whether SAMTOR remains associated with the inhibitory complex [43].

Methionine sensing by mTORC1 is not limited to SAMTOR. Protein arginine methyltransferase 1 (PRMT1) methylates NPRL2, thereby reducing GATOR1-mediated inhibition of mTORC1 under methionine-replete conditions [44]. SAMTOR and PRMT1 therefore respond to the same metabolic signal through distinct mechanisms: SAMTOR through direct ligand binding and PRMT1 through SAM-dependent protein methylation. This dual regulation provides a direct connection between methionine availability, methylation capacity, and mTORC1 signaling.

The metabolic consequence of reduced SAMTOR–mTORC1 signaling during methionine insufficiency is a shift away from anabolic processes toward metabolic conservation. Reduced mTORC1 activity suppresses protein and lipid synthesis and facilitates autophagy and intracellular nutrient recycling.

These signaling systems also interact. Amino acid insufficiency can suppress mTOR through a GCN2–FBXO22 pathway, in which GCN2 activation promotes mTOR ubiquitination [40]. This provides an additional point of convergence between the integrated stress response and mTORC1 signaling and supports the concept that amino acid sensing operates through an interconnected network rather than through isolated pathways.

Importantly, dietary methionine restriction does not necessarily suppress mTORC1 uniformly across tissues. Intracellular SAM concentrations also depend on methionine transport, methionine adenosyl transferase activity, remethylation, and transmethylation demand, while mTORC1 simultaneously integrates insulin, energy status, and other amino acids. Consistent with this complexity, recent evidence indicates that prolonged dietary methionine restriction can preserve skeletal-muscle protein synthesis and resistance exercise-induced mTORC1 activation [45]. These findings emphasize that systemic methionine restriction cannot be translated directly into a predictable change in mTORC1 activity in every tissue.

Altogether, the SAM–SAMTOR–GATOR1/KICSTOR–mTORC1 axis provides a direct molecular mechanism through which cells couple methionine availability to anabolic activity. This pathway is reinforced by SAM-dependent PRMT1 signaling and intersects with GCN2-dependent amino acid stress responses. Rather than acting as an isolated methionine sensor, SAMTOR therefore functions within a broader nutrient-sensing network that integrates sulfur amino acid availability, methylation status, and cellular anabolic demand.

### 4.3. Integration of Sulfur Amino Acid Sensing Pathways

Cellular adaptation to amino acid availability is coordinated by interconnected nutrient- and stress-sensing pathways rather than by isolated signaling cascades. The GCN2–eIF2α–ATF4 pathway and the SAMTOR–mTORC1 axis respond to distinct biochemical consequences of amino acid insufficiency but converge to reduce anabolic activity, promote nutrient conservation, and support stress adaptation.

A major point of convergence is the regulation of protein synthesis. Amino acid insufficiency can activate GCN2 and increase eIF2α phosphorylation, thereby reducing global translation, while decreased mTORC1 activity suppresses growth-promoting protein synthesis through downstream effectors such as S6 kinase and 4E-binding proteins [36]. These complementary mechanisms reduce amino acid and energy demand while preserving selective synthesis of stress-responsive proteins, particularly ATF4.

ATF4 provides an important molecular bridge between the integrated stress response and mTORC1 signaling. Under amino acid stress, ATF4 can induce Sestrin2, which suppresses mTORC1 through the GATOR regulatory machinery [46]. This response is context-dependent, as cells with greater endogenous amino acid synthetic capacity may maintain mTORC1 activity despite ISR activation. Thus, the metabolic consequences of pathway crosstalk depend on both extracellular nutrient availability and intrinsic cellular metabolic capacity.

Recent studies indicate that the interaction between GCN2 and mTOR is bidirectional. mTOR can directly phosphorylate GCN2 and modulate its activity during amino acid starvation [47]. Conversely, amino acid depletion can activate a tRNA–GCN2–FBXO22 axis, promoting mTOR ubiquitination and contributing to suppression of anabolic signaling [40]. These findings demonstrate that GCN2 and mTOR participate in reciprocal regulatory loops rather than functioning as strictly parallel pathways.

This integration is particularly relevant during SAAR. Reduced methionine availability can decrease intracellular SAM and thereby favor SAMTOR-dependent inhibition of mTORC1, while amino acid stress can simultaneously activate ATF4-related signaling. However, canonical GCN2 activity is not obligatory for all responses to methionine or SAAR. In mice, major physiological responses to MR persisted in the absence of GCN2 and were associated with activation of a noncanonical PERK/NRF2 pathway [39]. Similarly, SAA deprivation can maintain hepatic ATF4 and FGF21 responses even when GCN2 is absent [4].

ATF4 therefore appears to function as a convergence point for several nutrient- and stress-sensitive signals. GCN2, PERK, and other eIF2α kinases can increase ATF4 activity, while mTORC1 status influences the anabolic environment in which this stress response operates. During SAAR, changes in sulfur metabolism and redox balance can therefore complement direct amino acid- and SAM-sensing mechanisms. The interaction between these pathways also coordinates autophagy and nutrient recycling. Reduced mTORC1 activity facilitates autophagy, whereas ATF4 enhances amino acid transport and metabolic adaptation. Together, these responses increase intracellular nutrient availability while reducing anabolic consumption, helping restore cellular homeostasis during moderate nutrient restriction [5].

Importantly, signaling responses are tissue- and context-dependent. Differences in methionine metabolism, SAM turnover, transsulfuration capacity, and anabolic demand mean that dietary SAAR does not produce identical GCN2 or mTORC1 responses in all tissues. This flexibility may help explain how systemic metabolic benefits can occur without uniform suppression of protein synthesis or growth signaling.

Taken together, the GCN2–eIF2α–ATF4 and SAMTOR–mTORC1 pathways should be viewed as interconnected components of a broader amino acid-sensing network. Their crosstalk enables cells to integrate information about amino acid availability, translational stress, SAM status, and redox balance and to coordinate protein synthesis, autophagy, nutrient acquisition, and metabolic adaptation during SAAR.

**Figure 1:**
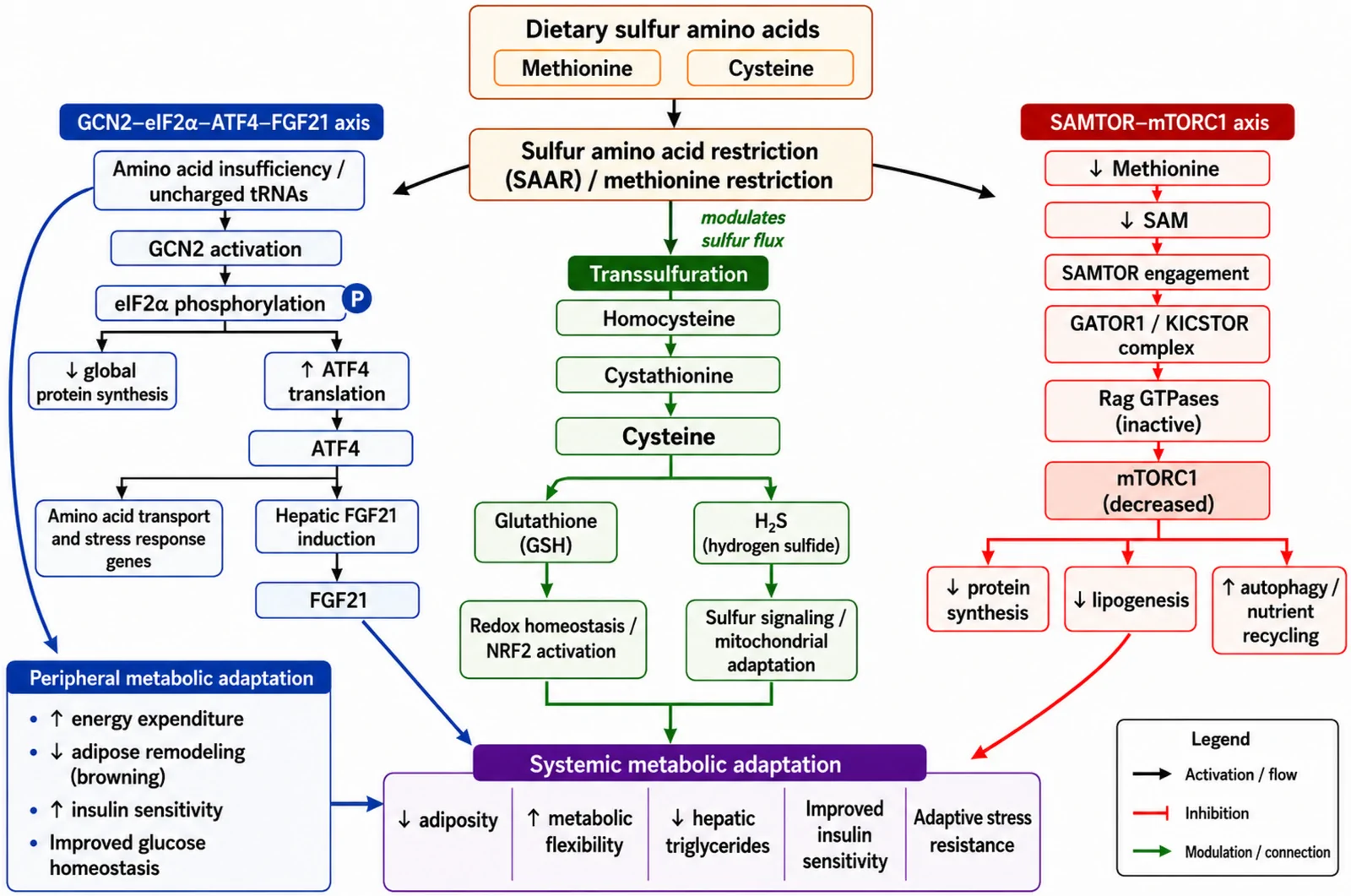
Integrated sulfur amino acid sensing and metabolic adaptation through the GCN2–ATF4–FGF21, transsulfuration, and SAMTOR–mTORC1 pathways. Reduced availability of methionine and cysteine activates complementary nutrient-sensing and metabolic pathways. Amino acid insufficiency can engage the GCN2–eIF2α–ATF4 pathway, leading to translational reprogramming, induction of hepatic FGF21, and peripheral metabolic adaptation. In parallel, reduced methionine availability lowers intracellular SAM, promoting SAMTOR-dependent inhibition of mTORC1 through the GATOR1/KICSTOR–Rag GTPase machinery, thereby decreasing anabolic processes and enhancing autophagy and nutrient recycling. SAAR also remodels transsulfuration and cysteine allocation toward GSH and H_2_S-dependent redox and mitochondrial signaling. Together, these pathways converge on systemic metabolic adaptation characterized by reduced adiposity and hepatic triglycerides, improved insulin sensitivity and metabolic flexibility, and enhanced stress resistance. Most downstream systemic metabolic outcomes illustrated in the figure are supported predominantly by cellular and animal studies, whereas direct evidence in humans remains more limited and heterogeneous. Abbreviations: ATF4, activating transcription factor 4; eIF2α, eukaryotic initiation factor 2 alpha; FGF21, fibroblast growth factor 21; GATOR1, GAP activity toward Rags 1; GCN2, general control nonderepressible 2; GSH, glutathione; H_2_S, hydrogen sulfide; KICSTOR, KPTN–ITFG2–C12orf66–SZT2 complex; mTORC1, mechanistic target of rapamycin complex 1; NRF2, nuclear factor erythroid 2-related factor 2; SAAR, sulfur amino acid restriction; SAM, S-adenosylmethionine; SAMTOR, S-adenosylmethionine sensor upstream of mTORC1.

The integrated sensing of SAA availability through the GCN2–eIF2α–ATF4, SAMTOR–mTORC1, and transsulfuration/redox pathways, together with their convergence on systemic metabolic adaptation, is summarized in Figure 1.

## 5. FGF21-Mediated Metabolic Adaptation to Sulfur Amino Acid Restriction

### 5.1. Hepatic FGF21 Induction During Sulfur Amino Acid Restriction

FGF21 is a hepatokine that plays a major role in systemic adaptation to nutritional stress. Although FGF21 can be expressed in several tissues, the liver is the principal source of circulating FGF21 during fasting, protein restriction, and selective amino acid deprivation [3,48]. In the context of SAAR, the liver therefore functions both as a major site of methionine metabolism and as an endocrine sensor capable of converting reduced amino acid availability into a systemic metabolic signal.

Hepatic FGF21 induction during amino acid restriction is closely linked to ATF4-centered stress signaling, although canonical GCN2 activation is not obligatory during SAAR. Experimental studies indicate that MR can maintain FGF21 induction through alternative stress-sensitive pathways, including PERK-related signaling [4,39,49]. Thus, FGF21 should be viewed as a convergent endocrine output of overlapping nutrient-sensing pathways rather than as the product of a single linear GCN2–ATF4 cascade.

The magnitude of FGF21 induction is influenced by the degree and type of amino acid restriction. Both selective MR and total protein restriction increase hepatic FGF21, although they are not mechanistically equivalent. Protein restriction reduces several essential amino acids simultaneously, whereas MR generates a more sulfur-specific signal.

Experimental studies nevertheless show that FGF21 is functionally important for many of the metabolic adaptations induced by protein or MR, including changes in energy balance, adiposity, and insulin sensitivity [7,50].

Redox-sensitive signaling may also modulate the hepatic FGF21 response. MR alters sulfur flux and glutathione metabolism, and GCN2-independent activation of PERK/NRF2 has been observed in experimental models [39]. However, liver-specific deletion of *Nfe2l2* does not abolish MR-induced FGF21 elevation, indicating that NRF2 is not essential for this endocrine response [51]. FGF21 induction should therefore be viewed as the product of a redundant nutrient-sensing network rather than of a single upstream pathway.

Human studies provide important translational support. Controlled protein restriction in healthy lean men increased circulating FGF21 and the energy intake required to maintain stable body weight [10]. Likewise, human SAAR interventions have demonstrated substantial metabolic remodeling, although the magnitude of the FGF21 response appears more variable than in rodent MR models. This variability likely reflects differences in baseline metabolic phenotype, dietary composition, degree of restriction, and protein source.

Importantly, hepatic FGF21 induction should be distinguished from its downstream actions. FGF21 secretion represents the endocrine output of nutrient sensing, whereas its effects on adipose tissue, energy expenditure, and thermogenesis depend on signaling through fibroblast growth factor receptors (FGFR)/β-Klotho in target tissue. Furthermore, not all consequences of MR are FGF21-dependent; several hepatic lipid responses persist in its absence [50].

### 5.2. FGF21 Regulation of Adipose Tissue, Energy Expenditure, and Thermogenesis

In rodent models, one of the most characteristic systemic effects of dietary MR is a reduction in adiposity accompanied by increased energy expenditure and thermogenic remodeling of adipose tissue. These effects frequently occur despite preserved or increased food intake, indicating altered energy partitioning rather than simple caloric restriction. FGF21 is a major mediator of this response in experimental animals. Human evidence supports FGF21 induction and metabolic remodeling during amino acid restriction, but pronounced thermogenic responses have not been consistently demonstrated [50,52].

FGF21 acts through fibroblast growth factor receptors in conjunction with the co-receptor β-Klotho. During MR, central nervous system signaling appears to be particularly important. Brain-specific deletion of β-Klotho markedly attenuates the increase in energy expenditure and adipose remodeling induced by MR, supporting a liver–FGF21–brain–sympathetic nervous system–adipose tissue axis [52]. Increased sympathetic activity subsequently promotes lipolysis, mitochondrial remodeling, and thermogenic gene expression in adipose tissue.

A prominent feature of MR is the browning of white adipose tissue, characterized by increased mitochondrial content and oxidative capacity. Uncoupling protein 1 (UCP1) contributes substantially to this response by promoting mitochondrial proton leak and heat production. In UCP1-deficient mice, the increase in energy expenditure and reduction in adiposity induced by MR are markedly attenuated, whereas improvement in insulin sensitivity is partly preserved [53]. This finding indicates that thermogenesis and insulin sensitization are mechanistically separable components of the MR phenotype.

In *FGF21*-deficient mice, MR fails to produce the normal increase in energy expenditure and adipose tissue remodeling, whereas some hepatic lipid-lowering effects remain intact [50]. These findings indicate that FGF21 is a major mediator of the thermogenic phenotype but is not required for all metabolic adaptations to MR.

MR also modifies adipokine secretion, typically increasing adiponectin and reducing leptin in association with lower adiposity [54]. These changes may further contribute to improved insulin sensitivity and lipid utilization, although they are not solely responsible for the metabolic phenotype.

Cysteine availability can also modify thermogenic responses. In mice, profound cysteine depletion induced extensive white adipose tissue browning, increased lipid utilization, and marked weight loss through sympathetic noradrenaline–β3-adrenergic signaling independently of both FGF21 and UCP1 [55]. Because this intervention represents extreme cysteine depletion rather than nutritionally controlled SAAR, its translational relevance is limited.

Human evidence is more limited. Controlled protein restriction increases circulating FGF21 and the energy requirement needed to maintain body weight [10], whereas direct SAAR trials have reported weight loss and metabolic remodeling without consistently reproducing the marked increases in resting energy expenditure observed in rodents [8]. Thus, human metabolic adaptation may depend more on altered substrate utilization and energy partitioning than on robust classical thermogenesis.

Preclinical evidence identifies FGF21-dependent central and adipose signaling as an important mediator of MR-induced thermogenesis and energy expenditure, whereas the corresponding response in humans appears substantially less pronounced. The broader influence of cysteine availability and the distinction between selective MR and total SAAR are discussed in Section 8.3.

### 5.3. FGF21-Mediated Regulation of Glucose and Lipid Metabolism

Preclinical studies show that dietary MR and broader SAAR improve glucose and lipid homeostasis through a combination of systemic endocrine effects and direct hepatic adaptations. In rodent models, these interventions enhance insulin sensitivity and glucose tolerance while reducing hepatic triglyceride accumulation [50,56,57]. Human intervention studies provide evidence of altered substrate utilization and metabolic signaling, but effects on glycemic control and hepatic lipid metabolism remain less consistently established.

FGF21 is a major mediator of the insulin-sensitizing response. In *Fgf21*-deficient mice, the improvement in insulin sensitivity induced by MR is markedly attenuated, whereas several hepatic lipid-lowering effects remain preserved. This indicates that systemic glucose regulation depends substantially on FGF21, while hepatic lipid metabolism also involves FGF21-independent mechanisms. This distinction between FGF21-dependent effects on insulin sensitivity and hepatic effects that are partially independent of FGF21 is directly supported by the study by Wanders et al. [50].

Improved hepatic insulin action appears to be an important component of the response. Recent SAAR studies show enhanced suppression of hepatic glucose production together with reduced liver triglyceride content [56,58]. These findings suggest that SAA restriction can simultaneously improve glucose control and hepatic lipid handling, two processes that are closely linked in insulin-resistant states.

MR also reduces hepatic lipid accumulation by limiting de novo lipogenesis and modifying substrate utilization. Reduced activity of lipogenic pathways, together with changes in fatty acid oxidation and metabolic flux, decreases the net storage of triglycerides in the liver [3,50,58]. Recent tracer-based studies further indicate that SAAR redistributes whole-body substrate utilization rather than simply reducing caloric intake, supporting a broader metabolic reprogramming toward lower lipid storage [58].

An important distinction must be made between nutritionally controlled MR and severe methionine deficiency. In experimental models, controlled MR can reduce hepatic lipid accumulation [50,56,58], whereas severe methionine deficiency, particularly when combined with choline deficiency, can impair phosphatidylcholine synthesis and very low-density lipoprotein (VLDL) export and thereby promote steatohepatitis. Thus, the metabolic response to sulfur amino acid restriction is strongly dose-dependent [13,50,58].

Cysteine availability further modifies these responses, reinforcing the need to distinguish selective MR from total SAAR [13,58]. This reinforces the need to distinguish selective MR from broader SAAR. This statement is well supported by Nichenametla et al., who directly compared methionine restriction, cysteine restriction, and broader SAAR [12,13].

Human evidence remains more limited than preclinical evidence. In adults with overweight or obesity, SAAR has been associated with greater weight loss and changes in leptin and ketone bodies, whereas effects on conventional glycemic and lipid parameters have been more modest [8]. Controlled feeding studies also indicate that methionine and total SAA restriction are feasible and biologically active in humans [12].

SAA restriction influences glucose and lipid metabolism through both FGF21-dependent and FGF21-independent mechanisms [50,56,57,58].

## 6. Transsulfuration, Redox Homeostasis, and Sulfur Signaling

### 6.1. Adaptive Regulation of the Transsulfuration Pathway

The transsulfuration pathway is dynamically regulated according to SAA availability, cellular cysteine demand, methylation status, and metabolic stress. Rather than operating at a fixed rate, it adjusts sulfur flux according to whether methionine must be conserved or whether cysteine production needs to be increased. This metabolic plasticity is particularly important during SAAR, when the balance between methionine conservation and cysteine synthesis becomes critical for maintaining cellular homeostasis [25,59,60].

A major regulator of transsulfuration is SAM. When methionine availability is high, increased SAM allosterically activates CBS while inhibiting MTHFR, thereby favoring the diversion of homocysteine toward transsulfuration rather than remethylation [25,59,61]. Conversely, when methionine availability decreases and SAM concentrations decline, this regulatory balance shifts toward conservation and regeneration of methionine. Through this mechanism, SAM functions not only as a methyl donor but also as an important metabolic signal coordinating the partitioning of homocysteine between competing pathways.

Cysteine availability also strongly influences transsulfuration activity. When extracellular cysteine or cystine becomes limited, cells with sufficient transsulfuration capacity can increase their dependence on endogenous cysteine synthesis from methionine-derived homocysteine. This adaptive mechanism can help preserve intracellular cysteine availability and sustain sulfur-dependent processes under conditions of extracellular cysteine limitation [62,63]. The importance of this response becomes particularly evident during oxidative and metabolic stress, when cellular demand for cysteine increases because of its requirement for glutathione synthesis and other sulfur-containing metabolites.

The relative restriction of methionine and cysteine therefore strongly determines the metabolic consequences of SAAR. Experimental studies demonstrate that cysteine restriction produces effects that cannot be fully reproduced by methionine restriction alone. In mice, cysteine restriction was identified as a major determinant of the anti-adiposity phenotype associated with SAAR and was linked to hepatic glutathione depletion, NRF2 activation, increased serinogenesis, and reduced substrate availability for glyceroneogenesis and triglyceride synthesis [13]. Importantly, the magnitude of these responses varies according to dietary fat content, sex, and age at the onset of restriction, further illustrating the context dependence of SAA metabolism.

Human feeding studies similarly indicate that selective methionine restriction and combined methionine–cysteine restriction are metabolically distinct interventions. In a controlled feeding study in healthy adults, total SAAR produced broader metabolic responses than selective methionine restriction, including reductions in body weight, insulin, leptin, low-density lipoprotein (LDL) cholesterol, and insulin-like growth factor 1 (IGF-1), despite both interventions increasing circulating FGF21 [12]. These findings emphasize that dietary cysteine can modify the physiological consequences of MR and that MR and total SAAR should not be considered metabolically equivalent.

Transsulfuration is also regulated at transcriptional and post-translational levels. CBS and CSE display tissue-specific patterns of expression and activity, with the liver representing a major site of SAA interconversion and glutathione synthesis [25,59,60]. Their activities can be modified by changes in redox state, phosphorylation, oligomerization, and subcellular localization, allowing sulfur metabolism to respond rapidly to changes in cellular conditions [64,65]. Recent evidence, for example, indicates that hypoxic stress can promote AMP-activated protein kinase (AMPK)-dependent phosphorylation of CSE, increasing persulfide and polysulfide formation and illustrating how cellular energy sensing can directly modulate sulfur metabolism [66].

Amino acid stress signaling provides another regulatory layer. Reduced extracellular amino acid availability activates adaptive transcriptional programs involving ATF4, which promotes the expression of genes involved in amino acid transport, biosynthesis, and redox maintenance [59,62]. In cells capable of transsulfuration, these responses can facilitate cysteine acquisition or endogenous cysteine production, thereby linking nutrient-sensing mechanisms with sulfur flux regulation. Transsulfuration therefore forms part of a broader metabolic network in which amino acid sensing, redox regulation, and sulfur allocation are coordinated rather than functioning as isolated pathways.

The dependence of both CBS and CSE on PLP further connects sulfur metabolism with vitamin B6 status. PLP insufficiency particularly affects CSE activity and can lead to accumulation of cystathionine, although compensatory mechanisms may preserve overall cysteine production and whole-body transsulfuration flux during moderate vitamin B6 restriction [66,67,68]. Controlled human studies have shown that short-term vitamin B6 restriction substantially lowers circulating PLP and increases cystathionine concentrations, while producing considerable interindividual variability in methionine-cycle and transsulfuration responses [67,68]. Thus, vitamin B6 status may influence the efficiency and flexibility of SAA metabolism even when global transsulfuration flux is not markedly reduced.

Importantly, the biological consequences of transsulfuration depend strongly on the severity of SAAR. Nutritionally controlled SAAR can activate compensatory metabolic and stress-response pathways while preserving essential sulfur-dependent functions. In contrast, profound cysteine depletion may exceed adaptive capacity. As discussed quantitatively in Section 8.3, these terms should be interpreted according to absolute methionine and total SAA intake rather than percentage restriction alone.

Recent experimental evidence shows that profound cysteine deficiency can rapidly deplete glutathione and coenzyme A, impair mitochondrial metabolism, activate integrated and oxidative stress responses, and produce extensive metabolic rewiring [69]. These findings highlight a fundamental distinction between adaptive SAAR and overt sulfur deficiency, which is especially important when considering clinical translation.

In conclusion, transsulfuration should be considered a regulated sulfur-allocation pathway rather than simply a route for homocysteine disposal. SAM availability, cysteine demand, nutrient-stress signaling, post-translational regulation, tissue-specific enzyme activity, and PLP status collectively determine whether sulfur is conserved or redirected toward cysteine and downstream sulfur-containing metabolites [25,59,64]. During SAAR, these regulatory mechanisms help define the boundary between beneficial metabolic adaptation and excessive SAA depletion.

### 6.2. Glutathione Homeostasis and NRF2-Mediated Redox Signaling

SAAR affects cellular redox homeostasis because methionine and cysteine availability directly influences reduced thiol pools and antioxidant capacity. However, SAAR does not simply cause GSH depletion. Nutritionally controlled MR may reduce oxidative damage and activate compensatory antioxidant responses, whereas profound cysteine deficiency can overwhelm these mechanisms and disrupt sulfur-dependent redox metabolism [2,13,69].

The glutathione system responds dynamically to sulfur amino acid availability. Redox capacity depends on GSH abundance, the GSH/GSSG couple, glutathione peroxidase and reductase activity, and NADPH availability [70,71]. Because cysteine is often rate-limiting for GSH synthesis, SAAR can alter both glutathione synthesis and recycling. Nevertheless, controlled MR may preserve antioxidant defense through compensatory redox adaptation [2].

NRF2 is a major regulator of this response. Under basal conditions, NRF2 is continuously ubiquitinated through Kelch-like ECH-associated protein 1 (KEAP1) and the cullin 3 (CUL3)-containing E3 ligase complex. Oxidative or electrophilic modification of redox-sensitive KEAP1 cysteines stabilizes NRF2, allowing nuclear accumulation and activation of antioxidant response element-dependent genes [72,73,74].

NRF2 regulates genes involved in glutathione synthesis and regeneration, including glutamate-cysteine ligase catalytic subunit *(GCLC)*, glutamate-cysteine ligase modifier subunit *(GCLM)*, and glutathione reductase *(GSR)*, as well as thioredoxin systems, detoxification pathways, heme metabolism, and NADPH-generating enzymes [71,74,75]. This broader metabolic role is particularly relevant during SAAR, when maintenance of redox balance requires coordinated regulation of sulfur metabolism and reducing-equivalent production.

Experimental MR rapidly induces hepatic stress-responsive transcriptional programs involving NRF2-associated genes, ATF4, extracellular signal-regulated kinases 1 and 2 (ERK1/2), and mTOR-linked signaling [10,49]. The interaction between ATF4 and NRF2 further links amino acid sensing to redox adaptation. ATF4 can promote NRF2 transcription, while NRF2 supports ATF4-driven adaptation by increasing cystine uptake and reinforcing thioredoxin-dependent defenses [76].

Cysteine restriction provides a clear example of this interaction. Experimental cysteine restriction lowers hepatic GSH and activates NRF2-responsive programs, producing metabolic effects distinct from MR alone [13]. Because cysteine is the immediate sulfur substrate for glutathione synthesis, its restriction imposes a particularly direct challenge to intracellular thiol homeostasis.

Nevertheless, NRF2 is not required for the entire systemic MR phenotype. In mice with liver-specific *Nfe2l2* deletion, MR-induced reductions in adiposity, increased energy expenditure, and FGF21 induction were largely preserved [51]. NRF2 should therefore be regarded as an important component of redox adaptation rather than the sole mediator of MR effects.

Redox remodeling also involves antioxidant systems beyond glutathione. In breast cancer models, MR transiently increased ROS, depleted GSH, reduced the GSH/GSSG ratio, and induced thioredoxin reductase 1 through NRF2- and ATF4-dependent mechanisms [77]. This supports the concept that MR can shift antioxidant reliance toward complementary thioredoxin-based defenses.

More broadly, glutathione peroxidases, peroxiredoxins, thioredoxin, thioredoxin reductases, catalase, and NADPH-generating enzymes function as an interconnected redox network [71,78]. Such redundancy may explain why moderate SAAR can alter glutathione metabolism without necessarily increasing oxidative injury.

A key concept is the threshold between adaptive redox signaling and sulfur deficiency. Low-to-moderate oxidative perturbations can activate protective signaling, whereas excessive oxidant production or insufficient antioxidant capacity leads to oxidative distress and macromolecular damage [79]. Moderate SAAR may therefore activate NRF2, ATF4, and complementary defenses, whereas severe cysteine depletion can exceed this adaptive capacity.

This distinction is supported by evidence showing that profound cysteine deficiency markedly depletes glutathione and coenzyme A, disrupts mitochondrial and intermediary metabolism, activates cellular stress responses, and causes rapid weight loss [69]. Such severe depletion represents functional sulfur deficiency rather than nutritionally moderate SAAR.

These dose-dependent effects have important translational implications. Beneficial restriction should induce metabolic and redox adaptation while preserving adequate pools of cysteine, glutathione, coenzyme A, and other essential sulfur metabolites. Excessive restriction may instead impair detoxification, redox buffering, mitochondrial function, and protein homeostasis. Future studies should therefore assess functional redox biomarkers, including GSH/GSSG status, sulfur metabolite profiles, and oxidative damage markers.

Human evidence remains limited. Controlled SAAR interventions indicate that moderate restriction can be implemented for several weeks without overt short-term toxicity, while producing substantial remodeling of circulating sulfur metabolites [8,12]. Sulfurome analyses have identified reductions in sulfate, cystathionine, sulfite, thiosulfate, taurine-related metabolites, and other sulfur compounds, together with signatures associated with adipose oxidative pathways and fat loss [9]. Whether similar adaptations consistently activate NRF2 or improve intracellular redox buffering in human tissues remains unknown.

Thus, SAAR induces a regulated redox adaptation rather than a uniform loss of antioxidant capacity. Moderate restriction can engage NRF2, ATF4, thioredoxin-dependent defenses, and NADPH-generating pathways, whereas severe cysteine depletion can overwhelm these mechanisms. The biological outcome therefore depends on the magnitude and duration of restriction and on tissue-specific capacity for compensatory redox adaptation.

### 6.3. Hydrogen Sulfide in Redox and Metabolic Adaptation

H_2_S is an important signaling molecule linking SAA metabolism to cellular adaptation. In SAAR, its relevance extends beyond sulfur flux because H_2_S can modulate protein function, mitochondrial metabolism, vascular responses, and redox signaling. The biochemical routes of H_2_S generation were described in Section 3.4; here, the focus is on its role as a metabolic signal during dietary restriction [80,81,82].

A major mechanism of H_2_S signaling is protein persulfidation, a reversible post-translational modification of cysteine residues that can alter enzyme activity, protein localization, transcriptional regulation, and susceptibility to oxidation. Persulfidation affects proteins involved in metabolism, redox control, inflammation, and mitochondrial function, supporting the concept of a broad sulfur-signaling network [81,82,83,84]. However, accurate detection and quantification of persulfidated proteins remain technically challenging [84].

Dietary restriction can reshape tissue-specific persulfidation profiles. Bithi et al. showed increased persulfidation in liver, kidney, skeletal muscle, and brain, with a distinct response in heart that depended substantially on cystathionine γ-lyase activity [85]. These findings provide a mechanistic link between nutrient availability and sulfur-based signaling.

H_2_S also interacts closely with mitochondrial metabolism. At physiological concentrations, sulfide can be oxidized through mitochondrial pathways and influence electron transfer and bioenergetics, whereas excessive H_2_S inhibits cytochrome c oxidase and impairs respiration [80,86,87,88]. Sulfide:quinone oxidoreductase (SQOR) acts as an important metabolic rheostat linking H_2_S oxidation to the coenzyme Q pool and mitochondrial electron transport [86,87].

Experimental evidence suggests that transsulfuration-dependent H_2_S production contributes to the beneficial effects of dietary restriction, including enhanced cellular stress resistance [89]. In humans, SAAR is associated with broad remodeling of sulfur-containing metabolites, although direct assessment of tissue H_2_S production remains technically difficult [90]. Thus, the contribution of H_2_S to the human SAAR phenotype remains biologically plausible but incompletely established.

H_2_S signaling also intersects with redox regulation. Persulfidation can protect redox-sensitive cysteine residues from irreversible oxidation and modulate the KEAP1–NRF2 pathway, thereby influencing antioxidant responses [81,82,84,91]. H_2_S-mediated persulfidation can also modify enzymes involved in sulfur and one-carbon metabolism, indicating that this modification may directly reshape metabolic flux [83].

H_2_S may additionally influence glucose and lipid metabolism through effects on mitochondria, vascular signaling, and insulin-responsive tissues [82,92,93]. However, these effects are strongly tissue- and concentration-dependent, making H_2_S one component of a broader adaptive network that also includes FGF21, ATF4, mTORC1, NRF2, and sulfur-dependent redox signaling [82].

Importantly, H_2_S exhibits a dose-dependent biological response. Physiological signaling may promote stress resistance and metabolic adaptation, whereas excessive sulfide accumulation is toxic. Mitochondrial sulfide oxidation therefore acts as an essential safeguard, allowing H_2_S to donate reducing equivalents to the respiratory chain at low concentrations while preventing inhibition of complex IV when sulfide levels rise [80,86,87,88].

Thus, H_2_S provides a mechanistic bridge between SAA metabolism and adaptive signaling through protein persulfidation, mitochondrial regulation, and redox-sensitive pathways. Its contribution to SAAR remains less firmly established than that of FGF21 or canonical nutrient-sensing mechanisms, particularly in humans. Future studies combining sulfur metabolomics, direct H_2_S flux measurements, and tissue-specific persulfidomics will be needed to define its contribution to beneficial versus maladaptive responses to SAAR [84,90].

### 6.4. Mitochondrial Redox Adaptation and Mitohormesis

Mitochondria are important targets of SAA restriction because changes in methionine and cysteine availability influence substrate oxidation, respiratory activity, redox balance, and cellular energy demand. Moderate MR does not simply suppress mitochondrial function; instead, it can promote a coordinated remodeling of oxidative metabolism that improves metabolic flexibility and stress resistance. This response is consistent with the concept of mito hormesis, whereby a mild mitochondrial stress activates adaptive mechanisms that enhance cellular resilience [2,94,95].

Mitochondrial reactive oxygen species (ROS) are central to this adaptive process. Although excessive ROS production causes oxidative damage, moderate increases can function as signaling molecules that trigger protective responses. The metabolic outcome therefore depends on the intensity and duration of the redox signal. During MR, altered SAA availability can modify mitochondrial substrate utilization and redox state sufficiently to induce adaptation without necessarily producing overt oxidative injury [2,96].

Experimental studies have demonstrated that MR increases mitochondrial biogenesis and oxidative capacity, particularly in white adipose tissue, while producing more tissue-specific effects in liver and skeletal muscle [3]. Adipose tissue undergoes especially pronounced remodeling, including increased mitochondrial content, enhanced oxidative metabolism, and greater uncoupled respiration. These adaptations are consistent with the increased energy expenditure and thermogenic phenotype associated with MR [94,95].

Increased mitochondrial uncoupling contributes to this response. By decreasing the efficiency with which the proton gradient is converted into ATP, uncoupling increases substrate oxidation and energy dissipation. UCP1 is an important mediator of this effect in thermogenic adipose tissue, although not all mitochondrial adaptations to sulfur amino acid restriction depend exclusively on UCP1 [39]. This indicates that mitochondrial remodeling includes both classical thermogenic mechanisms and broader changes in oxidative metabolism.

Human data provide important translational support. In healthy lean men, five weeks of dietary protein restriction increased circulating FGF21 and the energy intake required to maintain stable body weight. Proteomic analysis of adipose tissue revealed remodeling of mitochondrial electron transport proteins, while complementary experiments suggested that part of this response was FGF21-dependent [10]. Although protein restriction is not identical to selective SAAR, these findings support the concept that reduced amino acid availability can induce measurable mitochondrial adaptation in humans.

Another important consequence is improved metabolic flexibility, defined as the capacity to switch efficiently between lipid and carbohydrate oxidation according to nutrient availability. MR has been associated with enhanced substrate switching and increased oxidative metabolism under both fed and fasted conditions [94]. Such flexibility may contribute to improved metabolic health, particularly in obesity and insulin resistance, where mitochondrial substrate selection is frequently impaired.

The mitochondrial response is also strongly tissue-dependent. Adipose tissue can increase energy dissipation, whereas liver and skeletal muscle must preserve metabolic and functional demands specific to each organ. Thus, dietary SAAR should not be expected to produce uniform mitochondrial activation across all tissues [10,95]. Importantly, the adaptive response has a physiological limit. Moderate SAAR can generate a controlled mitochondrial stress that promotes metabolic remodeling and stress resistance, whereas severe cysteine deficiency can disrupt glutathione and coenzyme A availability and produce major metabolic stress [69]. This distinction is central to the concept of mitohormesis: beneficial adaptation requires a stress signal strong enough to activate protective mechanisms but not severe enough to compromise essential mitochondrial and sulfur-dependent functions.

Thus, mitochondrial remodeling represents an important component of the metabolic response to SAAR. Changes in respiratory activity, uncoupling, substrate oxidation, and redox signaling can increase energy expenditure and metabolic flexibility while enhancing cellular resilience. These adaptations appear to interact with FGF21-dependent endocrine signaling but are also shaped by tissue-specific sulfur metabolism and the severity of nutrient restriction. The transition from moderate, adaptive mitochondrial stress to severe metabolic dysfunction is therefore likely to be a key determinant of the biological effects of SAAR.

**Figure 2:**
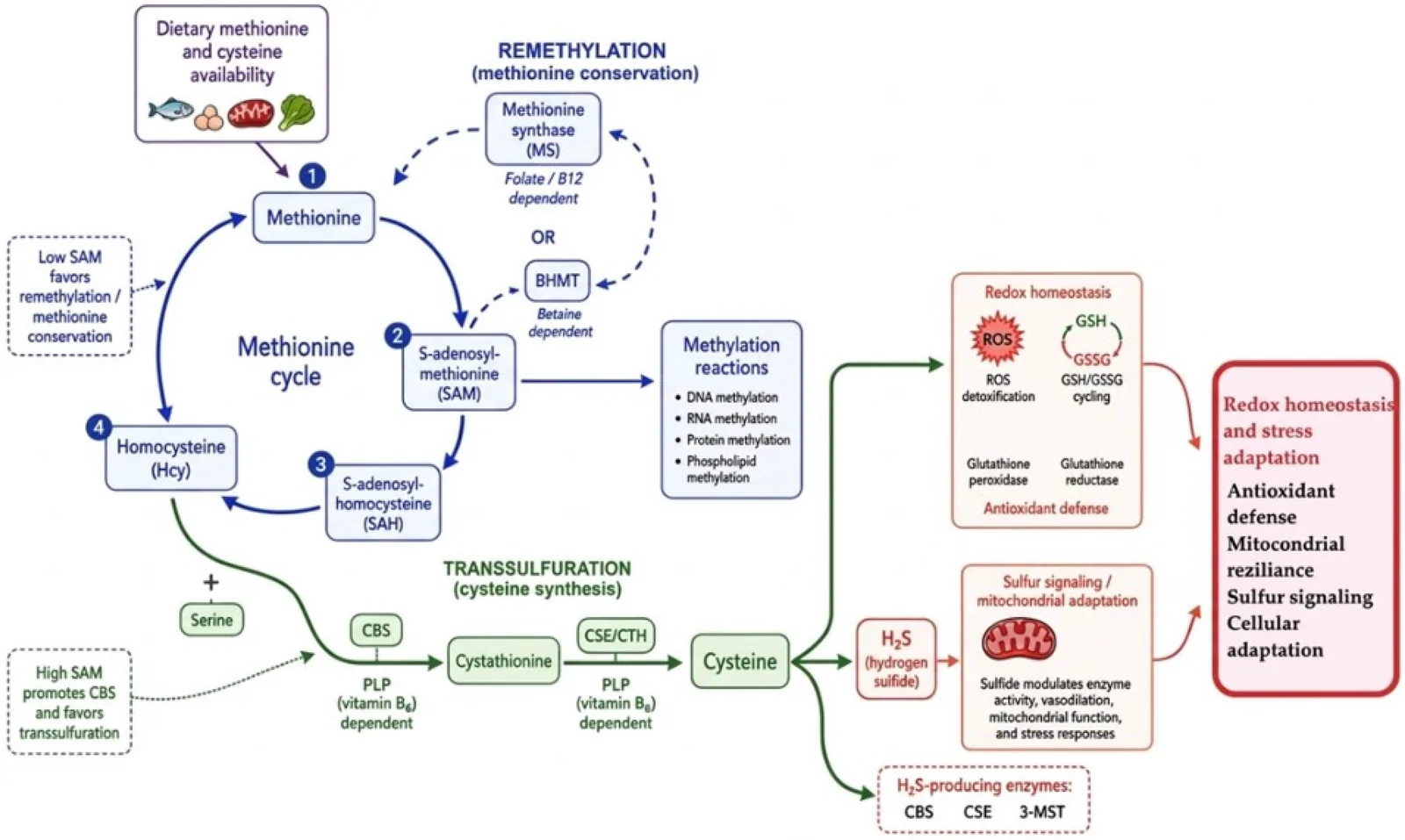
Integration of methionine metabolism, transsulfuration, and redox homeostasis. Dietary methionine enters the methionine cycle and is converted to SAM, which supports cellular methylation reactions and is subsequently converted to SAH and homocysteine. Homocysteine can be remethylated to methionine through MS or BHMT, thereby conserving methionine, or can enter the transsulfuration pathway. In transsulfuration, CBS and CSE/CTH, both dependent on PLP, convert homocysteine-derived sulfur into cysteine. Cysteine subsequently supports glutathione-dependent antioxidant defense and H_2_S production, linking SAA metabolism with redox homeostasis, mitochondrial adaptation, sulfur signaling, and cellular stress responses. SAM availability contributes to the balance between remethylation and transsulfuration. Abbreviations: BHMT, betaine-homocysteine methyltransferase; CBS, cystathionine β-synthase; CSE/CTH, cystathionine γ-lyase; GSH, reduced glutathione; GSSG, oxidized glutathione; Hcy, homocysteine; H_2_S, hydrogen sulfide; MS, methionine synthase; PLP, pyridoxal-5′-phosphate; ROS, reactive oxygen species; SAH, S-adenosylhomocysteine; SAM, S-adenosylmethionine.

The metabolic integration of methionine recycling, transsulfuration, cysteine utilization, and redox regulation is illustrated in Figure 2.

## 7. Sulfur Amino Acid Restriction in Metabolic Health and Disease

### 7.1. Sulfur Amino Acid Restriction in Obesity and Insulin Resistance

Obesity is characterized by excessive adipose tissue accumulation, adipocyte hypertrophy, altered adipokine secretion, chronic low-grade inflammation, ectopic lipid deposition, and impaired insulin signaling [97,98,99,100]. Preclinical studies indicate that dietary MR and SAAR can improve several of these abnormalities even without caloric restriction. In rodent models, MR reduces body weight and adiposity, improves glucose tolerance, and enhances peripheral insulin sensitivity, particularly under high-fat feeding or metabolic dysfunction [39,57,101,102]. Methionine-restricted mice remain relatively resistant to high-fat diet-induced adiposity and insulin resistance despite increased food intake, indicating metabolic reprogramming rather than simple energy restriction [102].

Reduced adiposity in experimental models is accompanied by altered energy partitioning, adipose remodeling, and changes in adipokine and inflammatory signaling [39,102,103,104,105]. These effects involve both FGF21-dependent and FGF21-independent mechanisms and may also include modulation of adipose DICER signaling [105].

MR also attenuates obesity-associated inflammation. In high-fat-fed mice, it reduces inflammatory gene expression in white adipose tissue and liver and suppresses signal transducer and activator of transcription 3 (STAT3) signaling, effects that remain evident in *Fgf21*-deficient animals [105]. Thus, SAAR can combine endocrine effects on energy balance with local anti-inflammatory actions.

Improved insulin sensitivity is another major component of the phenotype. MR enhances insulin receptor– protein kinase B (Akt) signaling and improves glucose homeostasis, sometimes before substantial weight loss occurs [57,101,106]. In adult mice, MR also reverses age-related adiposity and glucose intolerance and restores insulin responsiveness toward younger levels [103].

FGF21 contributes importantly to the insulin-sensitizing response to MR, although some hepatic lipid-lowering effects remain FGF21-independent [39,107]. Sex-dependent differences in FGF21 and adipose responses have also been reported [107,108].

These sex differences may have translational relevance because FGF21 responsiveness, adipose distribution, sex hormones, and baseline insulin sensitivity can modify the response to SAAR [54,108]. Pre-existing metabolic phenotype is also important: in *ob/ob* mice, adipose responses to MR were attenuated despite preservation of several hepatic adaptations [54].

Cysteine availability also modifies the anti-obesity phenotype. Experimental cysteine depletion can markedly alter adipose browning and energy expenditure, while cystine repletion reverses several SAAR-induced metabolic effects [55,58]. Because these findings derive from highly restrictive experimental models, their direct clinical relevance is limited.

Recent work has further clarified the mechanisms underlying improved glucose homeostasis. SAAR enhances whole-body glucose regulation, increases hepatic insulin responsiveness, and lowers hepatic triglycerides while stimulating de novo serine synthesis through the phosphoglycerate dehydrogenase–phosphoserine aminotransferase 1 (PHGDH–PSAT1) pathway [56]. Hepatocyte-specific loss of PSAT1 blunts the improvement in glucose tolerance but largely preserves reductions in adiposity and hepatic triglycerides, indicating that glucose and lipid adaptations can be mechanistically dissociated.

Alterations in glutathione metabolism may also contribute. In obese mice, SAAR lowers hepatic GSH while increasing NRF2, PHGDH, and serine concentrations, together with reductions in hepatic lipid droplets and fat mass; N-acetylcysteine reverses several of these changes [109].

SAAR also modifies substrate utilization in skeletal muscle. Increased lipid oxidation and fatty-acid utilization have been associated with improved exercise capacity, further supporting a coordinated multi-organ metabolic response [110].

Human evidence remains substantially more limited than the preclinical evidence. In adults with overweight or obesity, eight weeks of dietary SAAR produced greater weight loss than a higher-SAA control diet and was associated with lower leptin and increased ketone bodies [8]. Metabolomic analysis further identified plasma and urinary sulfur-metabolite changes associated with loss of body weight and android fat mass and with adipose expression of oxidative and β-oxidation genes [9]. However, the marked increases in energy expenditure commonly observed in rodent MR models were not reproduced, emphasizing important species and dietary-context differences.

Overall, preclinical evidence indicates that MR and SAAR can reduce adiposity, attenuate obesity-associated inflammation, and improve insulin responsiveness through both FGF21-dependent and FGF21-independent mechanisms. Human intervention studies provide preliminary support for effects on body weight, adiposity-related biomarkers, and substrate metabolism, but the magnitude and consistency of these responses are substantially more limited than in experimental models [54,104,108,110].

### 7.2. Sulfur Amino Acid Restriction in Type 2 Diabetes and Metabolic Syndrome

Type 2 diabetes (T2DM) and metabolic syndrome (MetS) arise from the convergence of insulin resistance, impaired glucose regulation, central adiposity, dyslipidemia, hepatic lipid accumulation, and chronic low-grade inflammation. Because SAAR simultaneously influences several of these processes, it has emerged as a potential nutritional strategy for improving the broader cardiometabolic phenotype rather than targeting a single metabolic abnormality. Experimental studies consistently indicate that MR improves glucose tolerance and insulin sensitivity and can delay progression toward overt diabetes in metabolically susceptible animals [50,56,57,101,102,106].

Particularly compelling evidence comes from New Zealand Obese mice, a polygenic model of obesity and T2DM. In this model, dietary MR prevented the development of hyperglycemia despite continued exposure to a high-fat diet and was associated with improved insulin sensitivity, increased adiponectin, elevated FGF21, reduced hepatic triglyceride accumulation, and enhanced hepatic insulin signaling [57,101,106]. These findings suggest that reduced methionine availability can interrupt the transition from obesity-associated insulin resistance to overt diabetes by acting simultaneously on hepatic and peripheral metabolism.

More recent studies extend this concept from selective MR to broader SAAR. In 2026, Ortega et al. demonstrated that SAAR improved whole-body glucose homeostasis, enhanced hepatic insulin action, and reduced liver triglyceride concentrations [56]. The simultaneous improvement of hepatic glucose regulation and lipid accumulation is highly relevant to MetS, because hepatic insulin resistance and steatosis often reinforce one another. These data support the view that sulfur amino acid availability influences an integrated metabolic network involving glucose production, lipid storage, and insulin responsiveness rather than isolated pathways [56,58].

These metabolic effects likely result from the convergence of endocrine, hepatic, adipose, and nutrient-sensing mechanisms, rather than from a single dominant pathway [101,103,105,106]. Thus, the potential benefit of SAAR in T2DM and MetS likely results from the convergence of several mechanisms already discussed in previous sections rather than from a single dominant pathway.

The relationship between sulfur amino acid intake and human cardiometabolic risk is also supported by observational evidence. Analysis of Third National Health and Nutrition Examination Survey (NHANES III) data showed that higher dietary SAA intake was associated with a less favorable cardiometabolic risk profile, including several components relevant to MetS [111]. Prospective epidemiological evidence further indicates that higher habitual intake of total sulfur amino acids and methionine may be associated with increased risk of developing T2DM [112]. Circulating SAA profiles have also been linked to future diabetes risk, with plasma total cysteine emerging as a potential metabolic correlate of incident diabetes [13]. Such associations cannot establish causality and may partly reflect broader differences in dietary patterns, particularly the contribution of animal versus plant proteins [112,113]. Nevertheless, they provide population-level support for investigating whether moderation of SAAs intake could improve cardiometabolic health.

Interventional human evidence is still limited. In adults with overweight or obesity, eight weeks of SAAR produced greater weight loss than a higher-SAA control intervention and was associated with decreased leptin and increased ketone bodies. A subsequent metabolomic analysis demonstrated substantial remodeling of the plasma and urinary sulfur metabolome, with metabolic signatures associated with loss of body weight and android fat mass and with adipose lipolytic gene expression [9]. However, the intervention did not establish that SAAR can directly reverse T2DM or MetS, and effects on conventional glycemic endpoints were less pronounced than those observed in experimental models.

This distinction is important for clinical translation. Current human data support metabolic feasibility and biological activity, but not yet the use of SAAR as a therapeutic treatment for established diabetes. Longer controlled studies in individuals with prediabetes, T2DM, or MetS are required, ideally incorporating glycated hemoglobin A1c (HbA1c), continuous glucose monitoring, hyperinsulinemic–euglycemic clamps, hepatic fat imaging, lipid profiles, and inflammatory biomarkers. The influence of medication use, sex, age, protein quality, and baseline SAAs intake will also need to be considered [112,113,114].

Thus, strong preclinical evidence indicates that moderate SAAR can improve several interconnected abnormalities underlying T2DM and MetS, particularly insulin resistance, hepatic glucose dysregulation, adiposity, and ectopic lipid accumulation [56,57,101,102,106]. Human observational studies provide associative support, whereas direct intervention evidence remains preliminary [8,111,112,113].

### 7.3. Sulfur Amino Acid Restriction in MASLD and Hepatic Lipid Metabolism

MASLD is closely linked to obesity, insulin resistance, dyslipidemia, and excessive hepatic triglyceride accumulation [115]. Because SAAR influences hepatic lipid metabolism and systemic insulin sensitivity in experimental models, it has emerged as a potentially relevant nutritional strategy for MASLD.

Preclinical studies consistently show that dietary MR reduces hepatic lipid accumulation and modifies pathways regulating lipogenesis, fatty acid oxidation, and substrate partitioning [56,58,103,116,117].

The hepatic lipid-lowering effect of MR is only partly dependent on FGF21. In *Fgf21*-deficient mice, MR still suppresses several hepatic lipogenic responses despite attenuation of its thermogenic and insulin-sensitizing effects [50]. This suggests direct hepatic responses to reduced methionine availability, potentially involving SREBP1c, lipogenic enzymes, mTORC1, and sulfur- and methyl-donor metabolism [116,117].

Recent preclinical evidence further supports the relevance of SAAR to hepatic steatosis. In an experimental model, Ortega et al. showed that SAAR lowered hepatic triglycerides while improving hepatic insulin action and whole-body glucose homeostasis [56]. These findings provide mechanistic support for a potential role of SAAR in MASLD, although direct clinical evidence remains limited.

These effects are particularly relevant to MASLD, where hepatic insulin resistance and lipid accumulation are closely interconnected [56,58].

MR also suppresses enzymes involved in de novo lipogenesis, including stearoyl-CoA desaturase-1 (SCD1). However, reduced SCD1 expression alone does not explain the full metabolic phenotype. Rather, decreased lipogenesis together with altered fatty acid oxidation and substrate utilization likely contributes to reduced hepatic triglyceride storage [58,103,116,117].

An important distinction must be made between nutritionally controlled MR and the profound methionine/choline deficiency produced by MCD diets. Methionine- and choline-deficient (MCD) diets induce steatohepatitis, hepatic lipid accumulation, inflammation, oxidative injury, and fibrosis [118,119,120,121,122]. These effects result from severe impairment of phosphatidylcholine synthesis, VLDL export, and methyl-donor metabolism and are fundamentally different from nutritionally controlled MR. FGF21 also appears protective in this setting, as *Fgf21*-deficient mice develop more severe hepatic injury during MCD feeding [119].

This distinction highlights the importance of dietary dose and composition. Excessive methionine restriction may impair phosphatidylcholine synthesis and VLDL assembly, whereas nutritionally controlled restriction appears to reduce hepatic lipid synthesis without disrupting essential export pathways [118,120,121,122].

Cysteine availability may further modify the hepatic response. Total SAAR is not metabolically equivalent to selective MR because cysteine influences methionine requirements, transsulfuration, redox balance, and lipid metabolism. Nichenametla et al. showed that cysteine restriction produces metabolic effects distinct from methionine restriction alone, emphasizing the need to report methionine and cysteine contents separately [13,58].

Human evidence specifically addressing MASLD remains limited. In adults with overweight or obesity, eight weeks of SAAR produced greater weight loss and metabolic changes than a higher-SAA diet, while metabolomic analyses demonstrated substantial remodeling of sulfur-containing metabolites [8]. However, these studies were not designed specifically for MASLD and did not establish reductions in hepatic fat or fibrosis.

Future trials should therefore recruit patients with imaging-confirmed MASLD and assess hepatic fat, insulin sensitivity, lipidomics, FGF21, sulfur metabolites, liver enzymes, and fibrosis markers, while avoiding excessive restriction in individuals at risk of sarcopenia or malnutrition.

Overall, preclinical evidence indicates that moderate SAAR can reduce hepatic triglyceride accumulation, suppress lipogenic pathways, and improve hepatic insulin responsiveness through both FGF21-dependent and direct hepatic mechanisms [50,56,58,103]. Human evidence specific to MASLD remains very limited, and current intervention studies have not established comparable effects on hepatic steatosis or fibrosis. These potential benefits must also be clearly distinguished from the hepatic injury caused by severe methionine/choline deficiency [118,120,121,122]. At present, SAAR should therefore be regarded as a promising but clinically unvalidated nutritional strategy for MASLD.

### 7.4. Tissue-Specific Metabolic Adaptations to Sulfur Amino Acid Restriction

The metabolic effects of SAAR are strongly tissue-dependent. Although reduced methionine and cysteine availability produces a coordinated whole-body phenotype, individual organs respond according to their roles in nutrient sensing, substrate utilization, and energy homeostasis [4,13,58,104].

The liver acts as the principal metabolic sensor and endocrine coordinator of MR. Reduced methionine availability rapidly alters hepatic amino acid sensing and induces FGF21, while also modifying lipid synthesis, glucose production, sulfur metabolism, and insulin sensitivity [3,4,58]. Some hepatic effects are FGF21-independent, as reductions in hepatic lipid accumulation can persist despite disrupted FGF21 signaling [50].

White and brown adipose tissues are major downstream targets of this response. MR promotes adipose remodeling, increased oxidative metabolism, altered adipokine secretion, and greater energy dissipation [52,58,119]. Central FGF21 signaling contributes substantially to these effects, whereas reductions in white-adipose inflammatory signaling can occur partly independently of FGF21 [52,105].

Skeletal muscle shows a distinct response. MR improves glucose metabolism and insulin signaling in obese mice, while short-term restriction may preserve mitochondrial respiration and hydrogen peroxide metabolism [106,123,124]. More recent work shows that SAAR can increase resting lipid oxidation and endurance capacity through enhanced fatty acid turnover and β-oxidation involving vascular endothelial growth factor (VEGF) signaling and endothelial cluster of differentiation 36 (CD36)-dependent fatty acid delivery [110]. These exercise-related effects were independent of FGF21, and skeletal-muscle responses also varied with age of diet initiation and biological sex [125].

The brain functions mainly as an integrative target. Central FGF21 signaling contributes to energy expenditure and adipose remodeling [52]. In diabetic mice, MR has also been associated with improved brain glucose metabolism and cognitive function through an FGF21–FGFR1–AMPK–GLUT4 pathway, together with reduced neuroinflammatory and oxidative markers [2].

The kidney is less extensively studied but also appears responsive to MR. Experimental studies indicate altered renal substrate utilization, enhanced stress resistance, increased endogenous H_2_S production, and protection against kidney injury [126,127,128]. SAAR may also protect against renal ischemia–reperfusion injury through changes in cysteine catabolism and H_2_S metabolism [128].

Tissue specificity also extends to protein metabolism. MR reduces mixed and cytosolic protein synthesis in liver and skeletal muscle while largely preserving mitochondrial protein synthesis [129]. Biological sex and ATF4 status can further modify several physiological responses to SAAR [4].

Thus, the systemic phenotype of SAAR results from coordinated but non-identical responses across tissues. The liver senses the dietary signal and generates endocrine mediators; adipose tissue increases substrate turnover and energy dissipation; skeletal muscle adapts glucose and fatty acid utilization; and the brain integrates endocrine signals with systemic energy regulation [4,13,58,119]. Recent metabolic-flux studies further support this tissue-specific organization by demonstrating distinct changes in carbohydrate and lipid utilization across liver, adipose tissue, heart, brain, and other organs during SAAR [58].

**Figure 3:**
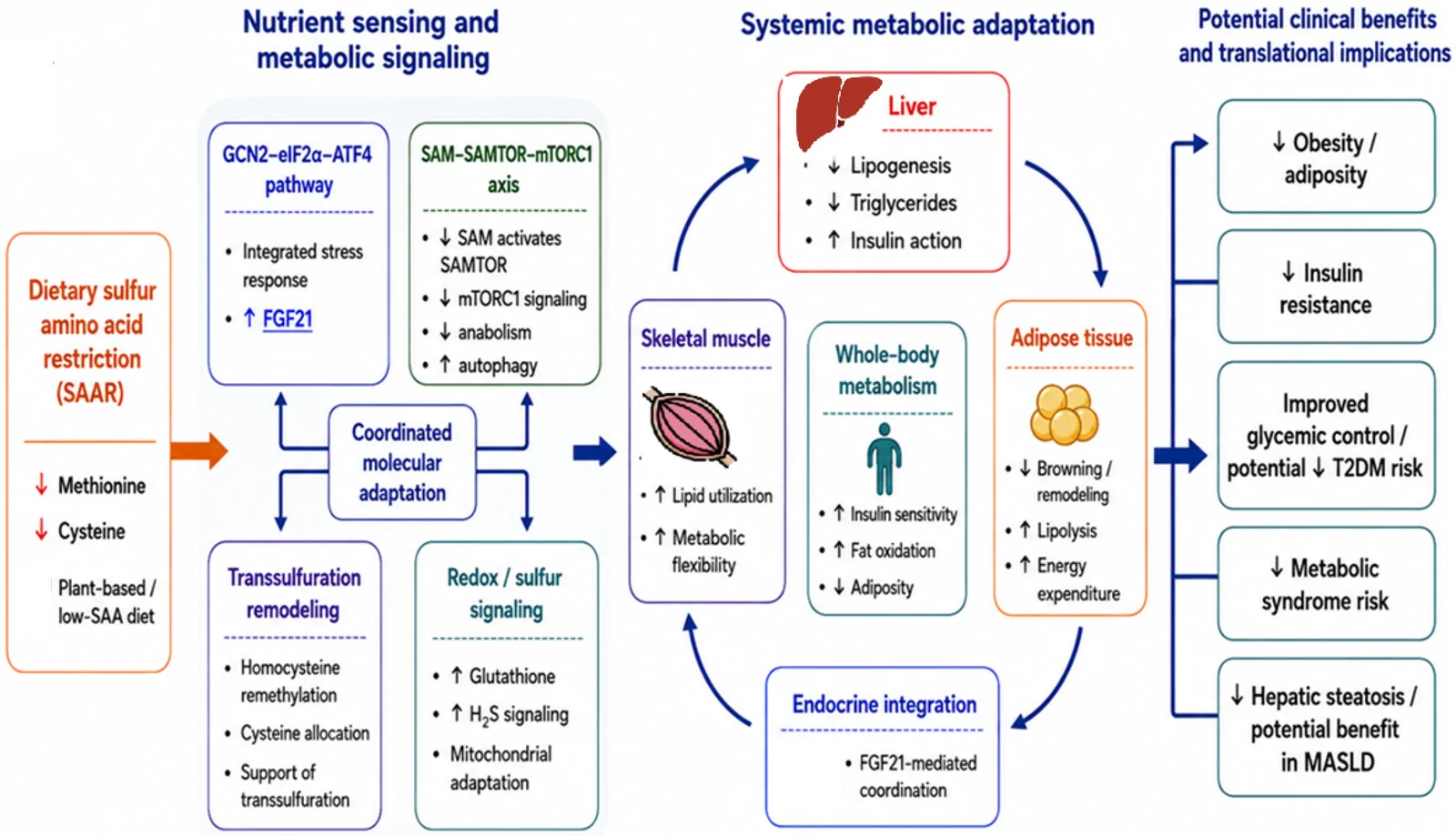
From dietary sulfur amino acid restriction to systemic metabolic adaptation and potential clinical benefits. Dietary SAAR, characterized by reduced methionine and cysteine availability, engages interconnected nutrient-sensing pathways including the GCN2–eIF2α–ATF4 axis and the SAM–SAMTOR–mTORC1 pathway, while also remodeling transsulfuration and redox/sulfur signaling. These molecular adaptations influence liver, adipose tissue, skeletal muscle, whole-body metabolism, and endocrine coordination through FGF21, leading to changes in lipid utilization, insulin sensitivity, energy expenditure, adiposity, hepatic triglyceride accumulation, and metabolic flexibility. Collectively, these responses produce robust metabolic benefits in preclinical models and may have translational relevance to obesity, insulin resistance, impaired glycemic control, metabolic syndrome, and hepatic steatosis. However, direct evidence for comparable clinical benefits in humans remains limited and requires confirmation in larger and longer intervention studies. These effects remain context-dependent and are influenced by the severity of restriction, cysteine availability, tissue specificity, and individual metabolic status. Abbreviations: ATF4, activating transcription factor 4; eIF2α, eukaryotic initiation factor 2 alpha; FGF21, fibroblast growth factor 21; GCN2, general control nonderepressible 2; H_2_S, hydrogen sulfide; MASLD, metabolic dysfunction-associated steatotic liver disease; mTORC1, mechanistic target of rapamycin complex 1; SAA, sulfur amino acids; SAAR, sulfur amino acid restriction; SAM, S-adenosylmethionine; SAMTOR, S-adenosylmethionine sensor upstream of mTORC1; T2DM, type 2 diabetes mellitus.

The integration of dietary SAAR with nutrient-sensing mechanisms, tissue-specific metabolic responses, and their potential clinical implications is summarized in Figure 3.

## 8. Translational Perspectives on Sulfur Amino Acid Restriction

### 8.1. Preclinical Evidence from Cellular and Animal Models

Cellular and animal studies provide the strongest mechanistic evidence for SAAR as a metabolic intervention. Reduced methionine or total SAA availability is associated with lower adiposity, improved glucose homeostasis and insulin sensitivity, altered hepatic lipid metabolism, increased energy expenditure, and activation of stress-response pathways. However, these effects vary with the degree of restriction, cysteine content, age, sex, background diet, and duration [55,57,101,102,130,131,132].

Cell-based studies have clarified mechanisms involving one-carbon metabolism, SAM-dependent processes, integrated stress signaling, and redox balance. During cystine limitation, transsulfuration can preserve intracellular cysteine and protect against oxidative and ferroptotic stress through the cysteine–GSH–GPX4 axis [133,134]. However, severe amino acid deprivation in vitro may not fully reflect moderate nutritional restriction in vivo.

Rodent studies show a relatively consistent phenotype of controlled MR, including reduced fat mass, improved glucose tolerance, and increased insulin responsiveness despite preserved or increased food intake [101,102,106,132]. In diabetes-prone New Zealand Obese (NZO) mice, MR prevented overt hyperglycemia and improved insulin sensitivity, while clamp studies confirmed enhanced whole-body and tissue-specific insulin action [57,106].

Responsiveness varies with biological context. Sex, age, and tissue type influence metabolic, mitochondrial, H_2_S, and redox responses to MR [130,131,135]. Dietary composition is also critical because many MR diets differ in cysteine content. Profound cysteine depletion can induce adipose browning, increased energy expenditure, and rapid weight loss, whereas more severe sulfur depletion can reduce glutathione and coenzyme A and induce metabolic stress [55,69]. Thus, profound sulfur deficiency should not be equated with nutritionally controlled SAAR.

Context-specific effects are also evident in cancer models. MR-induced sulfur deficiency has been reported to impair antitumor immunity through changes in gut microbial H_2_S production, whereas other models show enhanced cyclic GMP–AMP synthase (cGAS)-dependent or m^6^A-related antitumor immunity [13,136,137]. These findings underscore the importance of disease context and dietary design.

Controlled MR can improve glucose metabolism, body composition, and metabolic resilience, whereas excessive restriction may compromise sulfur-dependent processes [54,132]. Genetic and metabolic background also modifies the response; for example, leptin-deficient obese mouse model *(ob/ob)* mice show attenuated adipose responses despite preserved hepatic adaptations [54].

Overall, preclinical evidence supports SAA availability as an important regulator of adiposity, insulin sensitivity, hepatic metabolism, and stress adaptation, while identifying cysteine availability, sex, age, and disease context as major modifiers [54,101,102,132,135]. Differences in dietary formulation and restriction severity nevertheless limit direct extrapolation to humans.

Representative cellular and animal studies, together with their main outcomes, mechanisms, and limitations, are summarized in Table 1.

### 8.2. Clinical Evidence from Human Intervention Studies

Human evidence on MR and SAAR remains less extensive than the preclinical literature, but intervention studies indicate that dietary SAA manipulation is feasible and produces measurable metabolic responses. Overall, human responses appear less pronounced and more heterogeneous than those observed in experimental animals.

Reduced SAA availability can modify substrate metabolism and endocrine signaling, with reported changes in fat oxidation, FGF21, leptin, insulin-related parameters, lipid profiles, and sulfur-containing metabolites. However, responsiveness varies according to baseline metabolic status, habitual protein intake, sex, body composition, and degree of restriction [12,139,140,141,142,143].

Importantly, total SAAR is not metabolically equivalent to selective MR. Restriction of both methionine and cysteine can produce broader metabolic changes than MR alone, reflecting the cysteine-sparing effect and emphasizing the importance of total SAA availability [12,141,144,145]. This distinction is particularly relevant when translating rodent protocols that often combine very low methionine with little or no cysteine.

Energy expenditure also differs between species. Rodent MR commonly induces marked thermogenesis and hypermetabolism, whereas these effects are generally smaller in humans. Nevertheless, controlled protein restriction increases circulating FGF21 and the energy requirement needed to maintain body weight, supporting an effect of reduced amino acid availability on human energy metabolism [10,12,140].

Metabolomic studies further demonstrate systemic biochemical adaptation to SAAR. Dietary restriction alters the plasma and urinary sulfurome, including methionine-, cysteine-, and taurine-related metabolites, and specific sulfur-metabolite signatures have been associated with loss of body weight and android fat mass and with adipose metabolic responses [9]. Such profiles may therefore provide biomarkers of dietary adherence and metabolic responsiveness.

Current clinical evidence nevertheless has important limitations. Most studies involve small samples, short interventions, and participants who are healthy or overweight rather than patients with established metabolic disease [12,139,140,141]. Evidence in T2DM, metabolic syndrome, and MASLD remains scarce, and the optimal level of restriction that preserves protein adequacy and lean mass has not been established [142,143,144,145].

Thus, human studies demonstrate the feasibility and biological activity of SAAR but do not yet establish it as a therapeutic intervention. Larger and longer randomized trials are required to define dose–response relationships, responder phenotypes, nutritional safety, and clinically meaningful effects on insulin sensitivity, hepatic steatosis, and cardiometabolic risk.

The main characteristics, metabolic outcomes, biomarkers, and limitations of representative human intervention studies are summarized in Table 2.

### 8.3. Distinguishing Methionine, Sulfur Amino Acid, and Total Protein Restriction

MR, SAAR, and total protein restriction are frequently discussed together because all can activate nutrient-sensing pathways and induce overlapping metabolic adaptations. However, they are not metabolically equivalent interventions. Their effects differ according to which amino acids are limited, the extent of restriction, the availability of cysteine, and the degree to which overall protein intake is reduced [146,147]. For the purposes of this review, the terms moderate and severe restriction are used operationally rather than as formally established clinical categories. In adults, the Food and Agriculture Organization of the United Nations/World Health Organization/United Nations University (FAO/WHO/UNU) reference value for total sulfur amino acids is approximately 15 mg/kg/day as an average requirement, with a safe intake close to 19 mg/kg/day. Indicator amino acid oxidation studies have estimated a mean total SAA requirement of approximately 12.6 mg/kg/day and a population-safe intake of approximately 21 mg/kg/day when cysteine is absent [145]. Accordingly, for descriptive purposes within this review, human interventions providing total SAA intakes close to the recommended range (approximately 15–25 mg/kg/day), while maintaining adequate total protein intake, are considered to represent moderate SAAR. Importantly, these requirement and safe-intake estimates were established in a nutritional context and should not be interpreted as defining an optimal intake or clinically validated therapeutic target for metabolic intervention.

By contrast, severe restriction refers to interventions that markedly reduce methionine or total SAA availability below usual dietary exposure and, particularly, to experimental diets approaching very low methionine intakes or combined profound methionine/cysteine depletion. This distinction is intended to separate nutritionally controlled restriction from interventions that may approach functional sulfur amino acid deficiency.

Throughout this review, “moderate” restriction therefore refers to nutritionally controlled SAA reduction that remains close to established adult requirement or safe-intake ranges and preserves adequate total protein intake, whereas “severe” restriction refers to markedly lower methionine or total SAA availability, particularly when intake approaches levels that may compromise sulfur-dependent metabolic functions.

Selective MR lowers dietary methionine while maintaining cysteine intake. Because cysteine can spare methionine by reducing the need for transsulfuration-derived cysteine synthesis, the metabolic consequences of selective MR may be less pronounced than those produced when both sulfur amino acids are restricted [144,145,146]. This distinction is supported by human controlled-feeding data showing that total SAAR produced broader changes in body weight, circulating lipids, insulin, leptin, IGF-1, body temperature, and FGF21 than selective MR alone [12].

By contrast, SAAR restricts the combined availability of methionine and cysteine, thereby generating a stronger sulfur-specific nutritional signal. Experimental evidence indicates that cysteine restriction itself contributes independently to several metabolic effects previously attributed mainly to MR. In particular, cysteine availability influences adiposity, lipid metabolism, glutathione homeostasis, and thermogenic responses. This finding is important because many classical rodent “methionine-restricted” diets contain very little or no cysteine and therefore function physiologically as SAAR rather than as pure MR [13,147].

The distinction also has practical implications for translation. A diet containing reduced methionine but adequate cysteine may preserve methionine more effectively for essential functions, whereas simultaneous restriction of both sulfur amino acids places greater pressure on endogenous sulfur metabolism [25,144,145]. Moderate SAAR may therefore generate a stronger metabolic response, but excessive restriction also increases the risk of depleting sulfur-dependent metabolites and moving from adaptive nutrient stress toward deficiency. This dose dependence argues against treating all low-methionine dietary paradigms as interchangeable [25,147].

Total protein restriction is mechanistically broader still. Rather than selectively limiting sulfur amino acids, it reduces the availability of multiple essential and non-essential amino acids simultaneously. This can activate integrated amino acid stress responses and increase FGF21, but the initiating nutritional signal is less specific [3,7,146,148]. In mice, FGF21 is required for several metabolic effects of protein restriction, including reduced body weight and increased energy expenditure. In humans, controlled protein restriction likewise increases circulating FGF21 and raises the dietary energy requirement needed to maintain stable body weight, accompanied by mitochondrial remodeling in adipose tissue [7,10].

Although MR, SAAR, and total protein restriction can converge on shared adaptive pathways, including FGF21 and integrated amino acid-stress signaling, their initiating nutritional signals differ substantially. Clinically, the more important distinction is the absolute intake of methionine, cysteine, total SAA, and total protein and whether nutritional adequacy is maintained [3,25,146,148]. Consequently, protein restriction cannot be used as a direct surrogate for SAAR when assessing sulfur-specific mechanisms.

The interventions also differ in their potential clinical feasibility. Moderate SAAR can theoretically be achieved by modifying protein sources and reducing foods particularly rich in sulfur amino acids while maintaining total protein intake. The 2024 randomized trial in adults with overweight or obesity demonstrated that a relatively moderate SAAR intervention was feasible for eight weeks and produced greater weight loss together with changes in leptin and ketone bodies [8,147]. Total protein restriction, although potentially capable of producing similar endocrine adaptations, requires greater attention to preservation of lean mass and adequate intake of essential amino acids, particularly in older adults or individuals at risk of sarcopenia [144,148].

These differences also help explain why human responses are often less dramatic than those observed in classical rodent MR models. Rodent diets frequently employ very low methionine concentrations combined with minimal cysteine, creating a much stronger sulfur amino acid deficit than is generally acceptable in human nutrition [13,147]. Human interventions, in contrast, typically aim for moderate restriction while maintaining nutritional adequacy [12,144,145]. Direct comparisons across species therefore require careful consideration of both absolute methionine intake and total sulfur amino acid availability.

Thus, MR, total SAAR, and protein restriction should not be considered interchangeable, and future studies should report methionine, cysteine, total SAA, and total protein intake separately.

To integrate the mechanistic and translational evidence while avoiding repetition across sections, the principal pathways, predominant experimental support, available human evidence, and remaining uncertainties are summarized in Table 3.

### 8.4. Biomarkers of Response, Safety, and Precision Nutrition

Any future clinical translation of SAAR would require biomarkers that can identify dietary adherence, quantify biological response, and distinguish adaptive restriction from excessive sulfur amino acid depletion. At present, no single biomarker fulfills all of these functions. Instead, a multimarker approach combining circulating sulfur metabolites, endocrine signals, metabolic outcomes, and nutritional status is likely to be more informative [145,149,150].

Among candidate biomarkers, circulating FGF21 is particularly attractive because it responds to amino acid and protein restriction and reflects activation of the adaptive endocrine response. Human intervention studies have demonstrated increases in FGF21 during both SAAR and total protein restriction [10,12]. However, FGF21 lacks specificity for sulfur amino acid restriction because its concentrations are also influenced by fasting, protein intake, obesity, insulin resistance, and liver disease [151,152,153]. It should therefore be considered a marker of nutrient-stress responsiveness rather than a specific indicator of SAAR exposure.

Direct measurement of sulfur-containing metabolites may provide greater specificity. Methionine, total cysteine, cystathionine, taurine, sulfate, thiosulfate, and related metabolites change in response to dietary sulfur amino acid manipulation. Direct measurement of sulfur-containing metabolites may provide greater specificity. Methionine, total cysteine, cystathionine, taurine, sulfate, thiosulfate, and related metabolites change in response to dietary sulfur amino acid manipulation. Importantly, a distinct plasma and urinary “sulfurome” associated with loss of body weight and android fat mass has been identified, suggesting that combinations of sulfur metabolites may serve as biomarkers of both dietary exposure and metabolic responsiveness [9]. Plasma concentrations alone must nevertheless be interpreted cautiously because homeostatic mechanisms can maintain circulating cysteine despite marked changes in dietary intake and intracellular sulfur flux [140,144,154].

Additional metabolic markers may help characterize treatment response. Changes in leptin, insulin, lipid profiles, ketone bodies, body composition, hepatic fat, and indices of insulin sensitivity could be integrated with sulfur metabolomics to distinguish biochemical adherence from clinically meaningful benefit [8,150]. In individuals with metabolic disease, MRI-derived hepatic fat fraction, continuous glucose monitoring, or clamp-based insulin sensitivity may provide particularly relevant outcome measures. Thus, future biomarker strategies should separate three related but distinct questions: whether sulfur amino acid intake has actually been reduced, whether the expected molecular response has occurred, and whether this response has translated into metabolic improvement [144,150].

Safety is equally important because methionine and cysteine participate in protein synthesis, methyl-group metabolism, antioxidant defense, and numerous sulfur-dependent processes [149,155]. Human controlled-feeding studies indicate that moderate short-term MR or SAAR can be implemented without overt major toxicity, but existing trials are small and relatively short [12,140]. These findings cannot establish long-term safety, particularly in populations with higher protein requirements or impaired nutritional reserve [155,156,157].

The principal concern is therefore the distinction between controlled restriction and nutritional deficiency. Excessive restriction could compromise protein adequacy, lean mass preservation, glutathione-dependent defenses, methylation capacity, and other sulfur-dependent metabolic functions [149,155]. Particular caution is warranted in older adults, individuals with sarcopenia or frailty, pregnant or lactating women, growing children and adolescents, and patients with conditions associated with malnutrition [156,157,158,159]. In these populations, the potential metabolic advantages of aggressive SAAR may be outweighed by the consequences of inadequate essential amino acid or total protein intake. Accordingly, preservation of adequate total protein and essential amino acid intake, together with monitoring of body composition and lean mass, should be regarded as a prerequisite for any future clinical application of SAAR. This is particularly important in older adults, individuals with sarcopenia or frailty, pregnant or lactating women, growing children and adolescents, and patients with increased protein requirements or limited nutritional reserve. At present, low-SAA diets should not be interpreted as generally advisable therapeutic diets outside controlled nutritional or research settings.

Dietary composition also needs to be considered. Population studies suggest that lower SAA intake is often associated with dietary patterns containing a greater proportion of plant-derived proteins and with more favorable cardiometabolic profiles, but such observational findings do not establish that sulfur amino acids themselves are solely responsible for the association [111,154]. Changes in fiber, micronutrients, saturated fat, and overall diet quality can accompany shifts from animal to plant protein sources and must therefore be separated from sulfur-specific effects in future intervention studies [113,154].

These considerations strongly support a precision-nutrition approach rather than a universal target for sulfur amino acid intake. Individuals are likely to differ in their response according to habitual SAA intake, protein source, age, sex, adiposity, insulin sensitivity, liver function, renal function, genetic background, and potentially gut microbiota composition. Baseline circulating sulfur metabolites may also identify individuals with different metabolic phenotypes. Habitual dietary intake has been associated with variation in plasma methionine, cysteine, homocysteine, cystathionine, glutathione, and taurine, further illustrating that dietary exposure and endogenous sulfur metabolism interact at the individual level [113,144,146].

A future precision-SAAR strategy could therefore combine dietary assessment with baseline metabolic phenotyping and dynamic biomarkers of response [144,146]. Rather than prescribing a fixed degree of methionine or cysteine restriction, dietary sulfur amino acid intake could be adjusted to achieve a measurable adaptive response while maintaining adequate protein and sulfur-metabolite status. FGF21, sulfur-metabolite profiles, body composition, insulin sensitivity, and markers of nutritional adequacy could together help identify responders and determine whether further restriction is appropriate [8,10,144,150]. At present, however, these approaches remain investigational. No validated threshold for circulating FGF21 or individual sulfur-containing metabolites has been established to define an optimal or safe response to SAAR, and no clinically validated protocol currently exists for titrating methionine or total SAA intake on an individual basis. Accordingly, biomarker-guided or precision-SAAR strategies should be regarded as future research directions rather than currently applicable clinical tools.

Thus, biomarkers and safety considerations are central to the translation of SAAR from experimental models to clinical nutrition. FGF21 and sulfur metabolomics are promising indicators of biological response, but neither is sufficient alone [151,152,153]. Future clinical development will likely require integrated metabolic profiling combined with individualized assessment of protein requirements and disease risk; however, such an approach has not yet been clinically validated [155,156,157]. Such a precision-nutrition framework may eventually allow controlled sulfur amino acid restriction to be evaluated while preserving protein adequacy and lean mass and minimizing the risk of nutritional deficiency. Until long-term efficacy and safety are established, however, SAAR should remain a monitored experimental nutritional approach rather than an unrestricted dietary recommendation.

### 8.5. Current Controversies, Knowledge Gaps, and Future Directions

Despite substantial progress in understanding the metabolic effects of MR and SAAR, several major uncertainties limit their clinical translation. The most important issue is that MR, SAAR, and total protein restriction are frequently treated as interchangeable interventions, although they differ markedly in amino acid composition, metabolic signaling, and physiological consequences. Differences in dietary cysteine content are particularly important, because cysteine can spare methionine and independently modify redox balance, lipid metabolism, and thermogenic responses. Consequently, inconsistent dietary formulations remain a major source of heterogeneity across experimental studies [12,13].

A second unresolved question concerns the optimal degree of restriction. Much of the robust preclinical phenotype has been obtained using diets containing extremely low methionine concentrations, often together with little or no cysteine. Such interventions may generate metabolic effects that cannot be safely reproduced in humans. In contrast, nutritionally controlled restriction appears metabolically active but produces less dramatic responses. Recent evidence showing profound metabolic stress during extreme cysteine depletion further emphasizes that beneficial adaptation and nutritional deficiency exist on the same continuum [69]. Defining the range within which SAAR activates adaptive signaling without impairing essential sulfur-dependent functions is therefore a central research priority. Direct comparison of studies is further complicated by substantial heterogeneity in dietary protocols. Classical rodent MR diets frequently employ very low methionine concentrations, often together with little or no cysteine, whereas human interventions generally use substantially less severe restriction while preserving overall protein adequacy [12,13,139,140]. Differences in methionine and cysteine content, total protein intake, background diet, intervention duration, sex, and baseline metabolic phenotype may therefore contribute to apparently discordant findings across studies [12,13,54,108]. These protocol differences are particularly relevant when interpreting the much stronger FGF21 induction, thermogenic response, adiposity reduction, and hepatic lipid remodeling generally observed in rodents than in human intervention studies [8,10,12,50,52]. Consequently, quantitative dietary composition and species-specific physiology should be considered when assessing both mechanistic discrepancies and the translational relevance of preclinical findings.

The relative importance of individual molecular pathways also remains controversial. FGF21 is clearly involved in thermogenic and insulin-sensitizing responses to MR, yet several hepatic and anti-inflammatory effects persist in its absence [50,105]. Similarly, GCN2 and NRF2 contribute to aspects of the nutrient-stress response but are not universally required for the metabolic phenotype. These observations argue against a single linear mechanism and suggest substantial pathway redundancy and tissue specificity. Determining which pathways are necessary, sufficient, or merely associated with SAAR responses will require tissue-specific genetic models combined with metabolic flux analyses.

Another uncertainty concerns the translation of rodent thermogenesis to humans. Increased energy expenditure and UCP1-dependent adipose remodeling are prominent features of MR in mice, whereas human trials generally show much smaller changes in resting energy expenditure [8,10,12]. Differences in brown adipose tissue abundance, sympathetic thermogenic capacity, body size, and environmental conditions further limit direct extrapolation between species.

The influence of sex, age, genetic background, and baseline metabolic status also requires greater attention. Experimental data demonstrate sex-specific and age-dependent responses to MR, while human studies suggest substantial interindividual variability. Older adults represent a particularly important population because they may simultaneously benefit from improved metabolic health yet face greater risks from reduced protein intake and loss of lean mass. Future studies should therefore move beyond average treatment effects and identify biological characteristics that predict benefit or vulnerability [8,10,12].

An additional emerging area concerns interactions with the gut microbiota. Sulfur-containing dietary substrates influence microbial sulfur metabolism and H_2_S production, while microbial composition may modify host responses to methionine availability. Experimental evidence indicates that methionine restriction can alter host–microbiota interactions in ways that may be beneficial in some contexts but detrimental in others, including impaired antitumor immunity under specific experimental conditions [136]. Whether microbiome composition contributes meaningfully to variability in metabolic responses to SAAR in humans remains unknown.

Methodological limitations also complicate interpretation. Most studies rely on circulating amino acid concentrations or static metabolite measurements, which may poorly reflect intracellular sulfur flux. Stable-isotope tracing, tissue metabolomics, and integrated flux analyses will be required to determine how methionine is partitioned among protein synthesis, methylation, remethylation, transsulfuration, and downstream sulfur metabolites during dietary restriction [8,58,160]. Such studies are particularly needed in humans, where tissue-specific metabolic information remains scarce.

Finally, it remains unclear whether the benefits attributed to SAAR are achievable through specific dietary patterns rather than precisely formulated experimental diets. Plant-forward diets generally provide lower sulfur amino acid exposure than many animal-protein-rich dietary patterns, but they also differ in fiber, fat quality, micronutrients, and phytochemicals [112,113]. Future clinical studies must therefore distinguish sulfur amino acid-specific effects from improvements attributable to overall dietary quality.

Thus, the field has moved from demonstrating that sulfur amino acid restriction produces metabolic effects to addressing the more difficult questions of dose, specificity, individual responsiveness, durability, and clinical relevance. Future studies should standardize reporting of methionine, cysteine, total SAA, total protein, and energy intake; incorporate both sexes and different age groups; use tissue-specific and flux-based biomarkers; and conduct longer randomized trials in metabolically defined populations [8,10,12,113]. Resolving these uncertainties will determine whether SAAR remains primarily an experimental model of metabolic adaptation or could eventually be developed as a component of precision nutrition.

## 9. Conclusions

Sulfur amino acids play a central role at the intersection of nutrient sensing, one-carbon metabolism, redox regulation, and systemic energy homeostasis. Methionine and cysteine availability modulates interconnected pathways including GCN2–eIF2α–ATF4, SAMTOR–mTORC1, FGF21 signaling, transsulfuration, glutathione metabolism, H_2_S signaling, and mitochondrial adaptation.

Preclinical evidence consistently shows that moderate MR or SAAR can reduce adiposity, improve insulin sensitivity and glucose homeostasis, and decrease hepatic lipid accumulation. These effects are mediated by multiple, partially overlapping mechanisms, with FGF21 playing a major role in thermogenic and insulin-sensitizing responses, while several hepatic and redox adaptations remain FGF21-independent.

Importantly, MR, SAAR, and total protein restriction are not metabolically equivalent. Cysteine availability and the severity of restriction strongly influence the metabolic response. Moderate restriction may promote adaptive and hormetic signaling, whereas excessive sulfur amino acid depletion can compromise glutathione, coenzyme A, protein adequacy, and other essential metabolic functions.

Human intervention studies confirm the feasibility and biological activity of SAAR, but clinical evidence remains limited compared with animal studies. Larger and longer randomized trials are needed to establish the optimal degree of restriction, long-term safety, and efficacy in obesity, T2DM, MetS, and MASLD.

In conclusion, SAAR represents a promising area of nutritional research linking specific amino acid availability to endocrine, redox, and metabolic adaptation. However, SAAR cannot currently be considered an established therapeutic dietary intervention for obesity, T2DM, MetS, or MASLD, because long-term efficacy, safety, optimal restriction levels, and effects on clinically meaningful outcomes remain undetermined. Its future clinical application will therefore require larger and longer randomized trials and will likely depend on a precision-nutrition approach that considers individual metabolic phenotype, age, sex, protein requirements, and sulfur amino acid status.

## Figures and Tables

**Table 1:** Preclinical Studies Investigating Metabolic Adaptation to Methionine and Sulfur Amino Acid Restriction.

Model	Intervention Design	Duration	Main Metabolic Effects	Molecular Mechanism	Key Limitation	References
Male Fischer 344 rats (F344)	MR diet: 0.17% methionine vs. 0.86% in control (~80% reduction); methionine was the sole dietary SAA source	Long-term	Reduced adiposity, improved glucose tolerance and insulin sensitivity	Altered nutrient partitioning and improved insulin responsiveness independent of caloric restriction	Older study; male rats only; molecular pathways incompletely characterized	Malloy et al., 2006 [138]
Male rats	MR diet: 0.17% methionine vs. 0.86% in control (~80% reduction); no added cysteine/cystine	Several weeks	Increased energy expenditure and metabolic flexibility, with enhanced uncoupled respiration	Enhanced mitochondrial uncoupling and substrate switching are accompanied by increased UCP1 expression in brown and white adipose tissue, reduced leptin levels, and increased adiponectin	Limited molecular characterization; rodent physiology	Hasek et al., 2010 [94]
Adult mice	MR diet: 0.172% methionine vs. 0.86% in control (~80% reduction); total amino acid content maintained by compensatory adjustment	Several weeks	Reduced adiposity, improved glucose tolerance and insulin sensitivity, and increased circulating FGF21	Increased hepatic FGF21 and eIF2α–ATF4 signaling, reduced hepatic lipogenic gene expression, and enhanced IR/Akt insulin signaling	Degree of restriction greater than usually achievable in humans	Lees et al., 2014 [101]
Model	Intervention design and dietary SAA composition	Duration	Main metabolic effects	Molecular mechanism	Key limitation	References
Mice with brain- or adipose-specific β-Klotho deletion	MR diet: 0.17% methionine vs. 0.86% in control (~80% reduction); cysteine: 0% in both diets; amino acid content: 14.1% wt/wt	Several weeks	Increased energy expenditure and adipose tissue remodeling, with attenuated effects following central nervous system β-Klotho deletion	Hepatic FGF21 acts on FGFR/β-Klotho signaling in the central nervous system, leading to sympathetic activation and subsequent thermogenic remodeling of adipose tissue	Tissue-specific knockout model; human relevance of strong rodent thermogenesis uncertain	Forney et al., 2020 [52]
Liver-specific *Nfe2l2* knockout mice	MR diet: 0.17% methionine vs. 0.86% in control (~80% reduction); cysteine: 0% in both diets	Several weeks	MR-induced reductions in adiposity and increases in energy expenditure were largely preserved	Activation of NRF2-associated redox pathways, but hepatic NRF2 is not essential for FGF21 induction or the major systemic MR phenotype	Does not exclude NRF2 functions in extrahepatic tissues	Fang et al., 2021 [51]
Lean and obese mice	Cystine-free diet (100% dietary cystine removal) versus cystine-containing control, with cysteine/cystine repletion experiments	Short-term	Profound weight loss, increased lipid utilization, and extensive WAT browning	Sympathetic noradrenaline–β3-adrenergic signaling promotes adipose thermogenesis; effects reported to be FGF21- and UCP1-independent	Severe cysteine depletion; phenotype cannot be equated with nutritionally controlled dietary SAAR	Lee et al., 2025 [55]
Male C57BL/6J mice	High-fat diet with or without SAAR	6 weeks	Reduced adiposity and hepatic triglyceride levels, with improved hepatic insulin action and glucose homeostasis	Marked induction of hepatic de novo serine synthesis through PHGDH–PSAT1; hepatocyte PSAT1 is required for the glucose, but not lipid, adaptations to SAAR; reduced lysine lactoylation may enhance PHGDH activity	Male mice only; relative contribution of methionine versus cysteine restriction remains unresolved	Ortega et al., 2026 [56]

Abbreviations: F344, Fischer 344 rat strain; C57BL/6J, C57BL/6J mouse strain; FGF21, fibroblast growth factor 21; GSH, glutathione; MR, methionine restriction; NRF2, nuclear factor erythroid 2-related factor 2; NZO, New Zealand Obese; PHGDH, phosphoglycerate dehydrogenase; PSAT1, phosphoserine aminotransferase 1; SAAR, sulfur amino acid restriction; SCD1, stearoyl-CoA desaturase 1; T2DM, type 2 diabetes mellitus; UCP1, uncoupling protein 1; WAT, white adipose tissue.

**Table 2:** Human Intervention Studies Evaluating Sulfur Amino Acid Restriction and Related Dietary Approaches.

Participants		Duration	Main Outcomes	Biomarkers/Metabolic Responses	Clinical Implications	Key Limitations	References
26 adults with obesity and metabolic syndrome	Selective MR: ~2 mg methionine/kg/day versus ~35 mg/kg/day in the methionine-supplemented control (~94% reduction); cystine was maintained; target protein intake ~0.8 g/kg/day	16 weeks	Increased fat oxidation, reduced intrahepatic lipid content, with no additional effect on body weight or insulin sensitivity compared with the control group	Reduced plasma methionine and respiratory quotient, indicating a shift toward greater fat oxidation, with no increase in energy expenditure	First proof-of-concept that MR can modify fuel utilization in humans	Small sample; severe MR; cysteine was not restricted; limited effects on clinical endpoints	Plaisance et al., 2011 [139]
14 healthy, normal-weight adults	Low-SAA diet: total SAA 0.93 g/day in women and 1.19 g/day in men versus 5.75 and 6.01 g/day, respectively, in the high-SAA control diet (≈80–84% lower total SAA intake); intervention also differed in fatty-acid composition	7 days	No significant change in SCD activity indices; clear biochemical response to SAA reduction	Reduced plasma methionine and cystathionine and reduced urinary cysteine and taurine, with maintenance of circulating total cysteine; no significant differences in SCD activity indices.	Demonstrated rapid metabolic adaptation to SAAR and compensatory maintenance of circulating total cysteine	Very small sample; only 7 days; intervention simultaneously differed in fatty-acid composition, limiting attribution solely to SAAR	Olsen et al., 2018 [140]
20 women with overweight/obesity; 19 completed	Low Met/Cys: 1.6 g SAA/day (0.8 g Met + 0.8 g Cys); medium: 3.64 g/day (1.48 g Met + 2.16 g Cys); high: 5.6 g/day (2.12 g Met + 3.48 g Cys). Low and medium intakes corresponded to approximately 28% and 65% of the high-SAA intake, respectively	7 days	Modest effects on circulating markers; altered adipose lipid-metabolism gene expression	Increased FGF21, reduced plasma methionine and cystathionine, reduced urinary total cysteine, and increased adipose DGAT1 expression	Provided direct evidence that combined Met/Cys restriction is feasible and biologically active in overweight humans	Pilot trial; small sample; women only; short duration; insufficient power for clinical metabolic outcomes	Olsen et al., 2020 [141]
Participants	Intervention and SAA/protein intake	Duration	Main outcomes	Biomarkers/Metabolic responses	Clinical implications	Key limitations	References
20 healthy adults, 11 women/9 men	Controlled feeding with selective MetR or total SAAR: control, 30.1 mg Met + 30.1 mg Cys/kg/day; 70% MetR, 9.0 mg Met + 30.1 mg Cys/kg/day; 90% MetR, 3.0 mg Met + 30.1 mg Cys/kg/day; 50% SAAR, 15.0 mg Met + 15.0 mg Cys/kg/day; 65% SAAR, 10.4 mg Met + 10.4 mg Cys/kg/day	Three 4-week feeding periods, separated by washouts	SAAR reduced body weight, total cholesterol, LDL-C, insulin, leptin, uric acid, BUN, and IGF-1, while increasing body temperature; selective MetR produced fewer metabolic changes	Increased FGF21 with both interventions, a greater metabolic response with total SAAR, and reduced plasma methionine and cysteine with more pronounced SAAR	Suggests broader metabolic efficacy of combined Met/Cys restriction than selective MR, with short-term feasibility and no major safety signals	Small sample; healthy subjects; relatively short intervention periods; complex sequential feeding design	Richie et al., 2023 [12]
59 adults with overweight/obesity; 73% women	Plant-based SAAR diet: mean total SAA intake 19.2 mg/kg/day (~1.8 g/day in women; ~1.9 g/day in men) versus 62.1 mg/kg/day in controls (~5.4–5.5 g/day), corresponding to ~69% lower SAA intake; protein intake remained adequate	8 weeks	Greater weight loss with SAAR (approximately 20% greater than control), reduced leptin levels, increased ketone bodies, and no significant increase in resting metabolic rate	Reduced urinary sulfate excretion and SAA-related biomarkers, accompanied by metabolic remodeling of adipose tissue	Provides direct RCT evidence that a nutritionally feasible SAAR intervention at 19.2 mg/kg/day can influence body weight and metabolic phenotype in humans; however, this intake should not be interpreted as an established optimal therapeutic target.	Short duration; predominantly women; participants did not have established T2DM/MASLD; modest effects on several conventional metabolic endpoints	Olsen et al., 2024 [8]
59 adults with overweight/obesity	SAAR: 19.2 mg SAA/kg/day (~1.8–1.9 g/day) versus 62.1 mg/kg/day (~5.4–5.5 g/day) in controls (~69% lower SAA intake); secondary metabolomic analysis of the same 8-week RCT	8 weeks	Sulfur-metabolite signature predicted greater loss of body weight and android fat mass	Reduced sulfur metabolites, 69% lower urinary sulfur excretion, and a sulfurome signature linked to greater fat loss, scWAT oxidative activity	Identifies candidate metabolic biomarkers of response and adherence to SAAR	Secondary analysis of the same RCT; associations do not establish causal roles for individual sulfur metabolites	Olsen et al., 2024 [9]

Abbreviations: BUN, blood urea nitrogen; DGAT1, diacylglycerol O-acyltransferase 1; ETC, electron transport chain; FGF21, fibroblast growth factor 21; H_2_S, hydrogen sulfide; IGF-1, insulin-like growth factor 1; LDL-C, low-density lipoprotein cholesterol; MASLD, metabolic dysfunction-associated steatotic liver disease; Met, methionine; MR, methionine restriction; PUFA, polyunsaturated fatty acids; SAA, sulfur amino acids; SAAR, sulfur amino acid restriction; SCD, stearoyl-CoA desaturase; scWAT, subcutaneous white adipose tissue; SFA, saturated fatty acids; T2DM, type 2 diabetes mellitus; TCA, tricarboxylic acid. Where available, methionine, cysteine, total SAA, and protein intakes are reported using the quantitative values provided in the original publications. Percent reductions were calculated relative to the corresponding control diet.

**Table 3:** Mechanistic pathways and levels of evidence linking sulfur amino acid restriction to metabolic adaptation.

Mechanism/Outcome	Cellular Evidence	Animal Evidence	Observational Human Evidence	Randomized/Interventional Human Evidence	Major Unresolved Question
GCN2–eIF2α–ATF4 signaling	Amino acid insufficiency activates the integrated stress response and ATF4-dependent transcription; SAA deprivation can also engage GCN2-independent stress pathways [4,34,35,36,37,38,39]	Rodent studies confirm ATF4 activation during MR/SAAR, although GCN2 is not obligatory for the full metabolic response [4,39]	Direct evidence is very limited	Human tissue-level activation of GCN2–ATF4 during SAAR has not been clearly established	Which integrated stress-response pathways predominate during nutritionally feasible SAAR in humans?
FGF21 endocrine signaling	Amino acid stress promotes hepatic FGF21 expression through ATF4-related and additional stress-sensitive pathways [7,38,39,49]	Strong animal evidence indicates that FGF21 mediates increased energy expenditure, adipose remodeling, thermogenesis, and insulin sensitization during MR [50,52,53]	Circulating FGF21 is associated with nutritional and metabolic status, but is not specific for SAA restriction	Controlled protein restriction and SAAR/MR interventions increase circulating FGF21, although metabolic responses are less pronounced and heterogeneous [10,12]	Can FGF21 reliably identify individuals with a clinically meaningful response to SAAR?
SAMTOR–mTORC1 signaling	Strong cellular and molecular evidence indicates that SAM availability regulates mTORC1 through SAMTOR and the GATOR1/KICSTOR–Rag system [5,6,43]	Experimental evidence supports altered mTORC1-related nutrient signaling during methionine restriction	Not established	Not established	Does nutritionally feasible SAAR meaningfully alter SAMTOR–mTORC1 signaling in human tissues?
Cysteine availability and transsulfuration	Cysteine availability regulates glutathione synthesis, transsulfuration flux, sulfur allocation, and cellular stress responses [13,62,63,64,65,66,67,68,69]	Cysteine restriction modifies adiposity, lipid metabolism, thermogenesis, and redox homeostasis and can produce effects distinct from selective MR [13,55,69]	Human dietary SAA exposure is associated with circulating sulfur metabolites, but causal interpretation is limited	Controlled-feeding studies show that total SAAR produces broader metabolic responses than selective MR [12]	What degree of cysteine restriction maximizes metabolic adaptation without inducing sulfur deficiency?
Redox remodeling and NRF2 signaling	Reduced cysteine availability modifies GSH homeostasis and activates NRF2- and ATF4-related antioxidant responses [69,75,76,77]	Rodent studies demonstrate GSH depletion and NRF2-associated adaptation, although hepatic NRF2 is not required for the entire MR phenotype [13,51]	Human sulfur-metabolite profiles vary with dietary SAA exposure, but tissue-level redox responses remain poorly defined	Human SAAR alters circulating and urinary sulfur metabolites, but direct evidence for NRF2-mediated tissue adaptation is limited [9]	Which biomarkers distinguish beneficial redox adaptation from progression toward sulfur deficiency?
Thermogenesis and energy expenditure	Mechanistic studies support FGF21-, sympathetic-, β-adrenergic-, and UCP1-related pathways	Consistent increases in energy expenditure and adipose browning are observed in rodent MR models; extreme cysteine depletion can also induce FGF21- and UCP1-independent thermogenesis [50,52,53,55]	Direct observational evidence is limited	Human MR/SAAR studies generally show smaller or inconsistent changes in resting energy expenditure compared with rodents [8,10]	Are human metabolic benefits driven primarily by thermogenesis or by altered substrate utilization and energy partitioning?
Hepatic lipid remodeling	SAA restriction alters lipogenic pathways, serine synthesis, redox metabolism, and substrate handling	Consistent animal evidence indicates reduced hepatic triglyceride accumulation and altered lipogenesis in MR/SAAR models [50,56,58]	Lower dietary SAA exposure has been associated with more favorable metabolic profiles, but confounding by overall diet quality is substantial	Direct interventional evidence for reduction in hepatic fat in humans remains limited	Can clinically feasible SAAR reduce hepatic steatosis or fibrosis in MASLD?
Obesity, insulin resistance, and glucose homeostasis	Cellular studies support effects on insulin signaling, nutrient sensing, redox regulation, and lipid metabolism	Extensive rodent evidence demonstrates reduced adiposity and improved insulin sensitivity and glucose homeostasis [39,57,101,102,103,104,105,106]	Observational human studies suggest associations between lower SAA intake and more favorable cardiometabolic profiles, but causality is uncertain	Short-term human interventions show weight loss and metabolic biomarker changes, but evidence remains limited and heterogeneous [8,12]	Are these effects sustained and clinically meaningful in obesity, T2DM, and metabolic syndrome?
Selective MR versus total SAAR	Cellular studies demonstrate distinct consequences of methionine and cysteine availability for methylation, transsulfuration, and redox homeostasis	Animal studies show that cysteine availability markedly modifies the phenotype attributed to MR [13,55]	Observational data cannot reliably distinguish selective MR from total SAAR	Controlled feeding demonstrates broader metabolic responses with combined Met/Cys restriction than with selective MR [12]	What methionine-to-cysteine ratio provides the best balance between efficacy, feasibility, and nutritional safety?

Evidence descriptors in this table refer specifically to the strength and consistency of findings within the indicated experimental level and should not be interpreted as evidence of established clinical efficacy. Abbreviations: ATF4, activating transcription factor 4; eIF2α, eukaryotic translation initiation factor 2 alpha; FGF21, fibroblast growth factor 21; GCN2, general control nonderepressible 2; GSH, glutathione; MASLD, metabolic dysfunction-associated steatotic liver disease; MR, methionine restriction; mTORC1, mechanistic target of rapamycin complex 1; NRF2, nuclear factor erythroid 2-related factor 2; SAA, sulfur amino acid; SAAR, sulfur amino acid restriction; SAM, S-adenosylmethionine; SAMTOR, S-adenosylmethionine sensor upstream of mTORC1; UCP1, uncoupling protein 1.

## Data Availability

No new data were created or analyzed in this study. Data sharing is not applicable to this article.

## Abbreviations

The following abbreviations are used in this manuscript:

3-MST	3-mercaptopyruvate sulfur transferase
Akt	protein kinase B
AMPK	AMP-activated protein kinase
ATF4	activating transcription factor 4
BHMT	betaine-homocysteine methyltransferase
BUN	blood urea nitrogen
C57BL/6J	C57BL/6J mouse strain
CBS	cystathionine β-synthase
CD36	cluster of differentiation 36
CpG	cytosine–phosphate–guanine
CSE	cystathionine γ-lyase
CTH	cystathionine gamma-lyase gene
CUL3	cullin 3
DEPDC5	DEP domain-containing protein 5
DICER	endoribonuclease Dicer
DNA	deoxyribonucleic acid
*EIF2AK4*	GCN2 gene
eIF2α	eukaryotic initiation factor 2α
ERK1/2	extracellular signal-regulated kinases 1 and 2
ETC	electron transport chain
F344	Fischer 344 rat strain
FAO	Food and Agriculture Organization of the United Nations
FBXO22	F-box only protein 22
*Fgf21*	gene symbol for fibroblast growth factor 21
FGF21	fibroblast growth factor 21
FGFR1	fibroblast growth factor receptor 1
GATOR1	GTPase-activating protein activity toward Rags 1
GCL	glutamate-cysteine ligase
GCLC	glutamate-cysteine ligase catalytic subunit
GCLM	glutamate-cysteine ligase modifier subunit
GCN2–eIF2α–ATF4	general control nonderepressible 2–eukaryotic translation initiation factor 2 alpha–activating transcription factor 4 signaling pathway
GSH	glutathione
GSR	glutathione reductase
GSS	glutathione synthetase
GSSG	glutathione disulfide
GTP	guanosine triphosphate
HbA1c	glycated hemoglobin A1c
Hcy	homocysteine
IGF-1	insulin-like growth factor 1
ISR	integrated stress response
KEAP1	Kelch-like ECH-associated protein 1
KICSTOR	KPTN–ITFG2–C12orf66–SZT2-containing regulator of mTORC1
LDL	low-density lipoprotein
m6A	N6-methyladenosine
MASLD	metabolic dysfunction-associated steatitis liver disease
MAT	methionine adenosyl transferase
MAT1A	methionine adenosyl transferase 1A
MAT2A	methionine adenosyl transferase 2A
MCD	Methionine- and Choline-deficient
MetS	metabolic syndrome
MPST	3-mercaptopyruvate sulfur transferase gene
MR	methionine restriction
mRNA	messenger RNA
MTHFR	methylenetetrahydrofolate reductase
mTOR	mechanistic target of rapamycin
mTORC1	mechanistic target of rapamycin complex 1
NADPH	nicotinamide adenine dinucleotide phosphate
NHANES III	Third National Health and Nutrition Examination Survey
NPRL2 and NPRL3	nitrogen permease regulator 2 and nitrogen permease regulator 3
NRF2	nuclear factor erythroid 2-related factor 2
NZO	New Zealand Obese
*ob/ob*	leptin-deficient obese mouse model
PERK	protein kinase RNA-like endoplasmic reticulum kinase
PHGDH	phosphoglycerate dehydrogenase
PLP	pyridoxal-5′-phosphate
PRMT1	protein arginine methyltransferase 1
PSAT1	phosphoserine aminotransferase 1
PUFA	polyunsaturated fatty acids
Rag GTPases	Ras-related GTP-binding proteins
Rheb	Ras homolog enriched in brain
RNA	ribonucleic acid
ROS	reactive oxygen species
SAAR	sulfur amino acid restriction
SAA	sulfur amino acids
SAH	S-adenosylhomocysteine
SAM	S-adenosylmethionine
SAMTOR	S-adenosylmethionine sensor upstream of mTORC1
SCD1	stearoyl-CoA desaturase-1
scWAT	subcutaneous white adipose tissue
SFA	saturated fatty acids
SLC7A11	solute carrier family 7 member 11
SQOR	sulfide:quinone oxidoreductase
SREBP1c	sterol regulatory element-binding protein 1c
STAT3	signal transducer and activator of transcription 3
T2DM	type 2 diabetes mellitus
TCA	tricarboxylic acid
tRNA	transfer RNA
UCP1	uncoupling protein 1
UNU	United Nations University
VEGF	vascular endothelial growth factor
VLDL	very-low-density lipoproteins
WHO	World Health Organization
